# Establishment and validation of a machine learning-based predictive model for in-hospital mortality risk in acute myocardial infarction patients complicated with diabetes mellitus

**DOI:** 10.3389/fcvm.2026.1833723

**Published:** 2026-06-01

**Authors:** Lang Zeng, Yangchun Li, Fenglin Wu, Shikang Li, Chenshi Rao, Yao Zhang, Yonghong Zhang, Xuefeng Ding, Houxiang Hu, Rongchuan Yue

**Affiliations:** 1 Department of Cardiology, Affiliated Hospital of North Sichuan Medical College, Nanchong, China; 2 Department of Cardiology, Bazhong Central Hospital, Bazhong, China; 3 Department of Critical Care Medicine, Affiliated Hospital of North Sichuan Medical College, Nanchong, China; 4 Department of Cardiology, Chengdu Fifth People’s Hospital (The Second Clinical Medical College, Affiliated Fifth People’s Hospital of Chengdu University of Traditional Chinese Medicine), Chengdu, China

**Keywords:** acute myocardial infarction, diabetes mellitus, in-hospital mortality, machine learning, predictive model

## Abstract

**Objective:**

To develop and validate a machine learning-based (ML) predictive model for in-hospital mortality risk in patients with acute myocardial infarction (AMI) complicated by diabetes mellitus (DM).

**Methods:**

This retrospective study enrolled AMI patients with DM from the Affiliated Hospital of North Sichuan Medical College and the MIMIC-IV database. Common variables identified by both LASSO regression and the Boruta algorithm were selected as the final feature set. Utilizing the MIMIC-IV database, predictive models were constructed incorporating seven machine learning algorithms based on these common variables. The comprehensive performance of these models was evaluated through extensive metrics in both internal and external validation sets to identify the preferred model. Finally, the SHapley Additive exPlanations (SHAP) method was employed to quantitatively analyze and visually display the feature contributions of the preferred model.

**Results:**

Seven predictors were identified in this study through variable selection using two distinct methods, including heart rate, neutrophil count, monocyte count, neutrophil-to-lymphocyte ratio (NLR), serum albumin, total bilirubin, and urea nitrogen. Seven different ML models were built based on these predictors. Comprehensive performance evaluation across multiple metrics in both internal and external validation sets has shown that the XGBoost-based model achieved the numerically highest AUC and was selected as the preferred model. By employing the SHAP method for visual interpretation of this model, the interpretability and clinical credibility of the in-hospital mortality prediction model were significantly enhanced. This model can provide valuable auxiliary support in identifying high-risk patients and implementing early intervention measures.

**Conclusion:**

Interpretable machine learning models have been developed to predict in-hospital mortality risk in patients with AMI complicated by DM, providing insights into the influence of various features on the prediction outcome. Therefore, this model can serve as an exploratory and auxiliary risk stratification tool limited to clinical settings similar to our study cohorts, and it is not intended for generalized broad clinical application.

## Introduction

Cardiovascular disease (CVD) is not only one of the major challenges to global human health and survival but also the leading cause of death worldwide, claiming nearly 17.7 million lives annually, with mortality rates remaining persistently high over the long term (1–3). This places a substantial burden on public health systems. Acute myocardial infarction (AMI) refers to myocardial ischemia and necrosis caused by a sharp reduction or complete interruption of coronary blood flow. It is not only one of the most severe types of CVD but also a major cause of the increasing global cardiovascular mortality rate (4–6). In recent years, due to unhealthy and irregular lifestyle habits, the incidence of AMI has been gradually increasing, causing millions of deaths worldwide each year (7, 8). It is estimated that the in-hospital mortality rate for patients with AMI ranges from approximately 7% to 14% (9). Diabetes mellitus (DM), another major and escalating global health issue, has seen its prevalence surge from 108 million in 1980 to 422 million in 2014, and is projected to affect more than 592 million people by 2035 (10). The coexistence of DM and AMI presents an increasingly severe synergistic threat to global health, thereby imposing a substantial dual disease burden. DM is also a major risk factor for AMI. As a prevalent comorbidity in hospitalized AMI patients, DM manifests in approximately 30% of cases, and nearly half of the patients are in a pre-diabetic state either during hospitalization or after discharge (11, 12). In AMI patients with comorbid DM, coronary artery lesions often involve multiple vessels, with more diffuse lesions, leading to more severe myocardial damage and consequently increasing the risk of mortality (13). Compared with AMI patients without DM, those with comorbid diabetes have significantly higher in-hospital and one-year mortality rates, and their mortality risk continues to increase with longer duration of DM (14, 15). Previous studies have demonstrated that from the pre-reperfusion era, through the era of thrombolytic therapy, to the era of percutaneous coronary intervention (PCI), the in-hospital mortality rate of patients with AMI complicated by DM has generally remained twice that of non-diabetic patients (16). Therefore, reducing in-hospital mortality, improving quality of life, and enhancing survival rates represent critical challenges. Addressing these significant challenges will require more precise strategies to improve clinical outcomes.

Previous research on predictive models for AMI-related diseases has shown that the commonly used statistical methods are predominantly based on logistic regression and Cox proportional hazards regression models (17–19). In recent years, machine learning (ML) has gradually emerged in clinical practice and gained increasing attention. As a branch of artificial intelligence, ML is built upon computer technology and big data. With the advent of large medical databases, ML methods can significantly enhance the performance of predictive models (5). Previous studies have demonstrated that ML exhibits superior predictive value compared with traditional prediction models and can better organize and analyze massive patient data (20–24). Consequently, ML methods represent an important tool in medical research (25, 26). The limitations of traditional predictive models, such as their inability to accurately and efficiently parse nonlinear relationships within massive, high-dimensional medical data that often contain missing values, their reliance on parameter assumptions and linearity, and their limited capacity for examining high-order interactions, can be effectively addressed by ML algorithms (5, 9). Furthermore, ML encompasses a wide variety of algorithms, including Random Forest (RF), Decision Trees (DT), Support Vector Machines (SVM), and Multilayer Perceptron (MLP), among others (5, 27). Given the advantages of machine learning, we constructed and compared multiple models to identify the optimal one for predicting in-hospital mortality risk among AMI patients complicated by DM. This will provide clinicians with a basis to accurately assess their in-hospital mortality risk and prognosis.

## Study subjects and methods

### Data source

This study has a retrospective design. To guarantee the robustness of the findings, two distinct patient cohorts were analyzed. The first cohort was sourced from the Medical Information Mart for Intensive Care-IV (MIMIC-IV-3.1) database, which contains information on more than 70,000 intensive care unit admissions at Beth Israel Deaconess Medical Center in Boston, Massachusetts, USA, from 2008 to 2019. This database provides comprehensive patient data, including demographics, vital signs, laboratory results, comorbidities, and treatment information. After we completed the CITI program's “Data or Specimens Only Research” course and passed the examination (record ID: 65680883), we were granted access to this database to extract the information required for this study. Since MIMIC-IV-3.1 is a publicly accessible, de-identified, and anonymized database, its analysis was exempt from the requirements of obtaining informed consent from study participants and additional ethical approval from an Institutional Review Board. The second cohort was selected from the integrated systems of the Affiliated Hospital of North Sichuan Medical College, including the Hospital Information System, Laboratory Information Management System, and Electronic Medical Record Systems. A total of 560 patients diagnosed with AMI complicated by diabetes between January 2021 and April 2025 were initially screened. This study was conducted in accordance with the ethical principles of the Declaration of Helsinki and was approved by the Ethics Committee of the Affiliated Hospital of North Sichuan Medical College (Ethics Approval Number: 2026ER111-1). Because this study only involves a retrospective analysis of patients' existing clinical data, the ethics committee waived the requirement for informed consent.

### Study population and design

The inclusion criteria were as follows:(1) age >18 years; (2) meeting the diagnostic criteria for AMI (28); (3) Diagnostic criteria for DM: 1. Previously diagnosed with DM and currently receiving treatment with glucose-lowering medications or insulin; 2. Meeting the diagnostic criteria outlined in the 2020 American Diabetes Association (ADA) guidelines (29).

The exclusion criteria were as follows: (1) history of chronic myocardial infarction (MI); (2) history of malignant tumor; (3) hospitalization duration <24 h.

**Figure 1:**
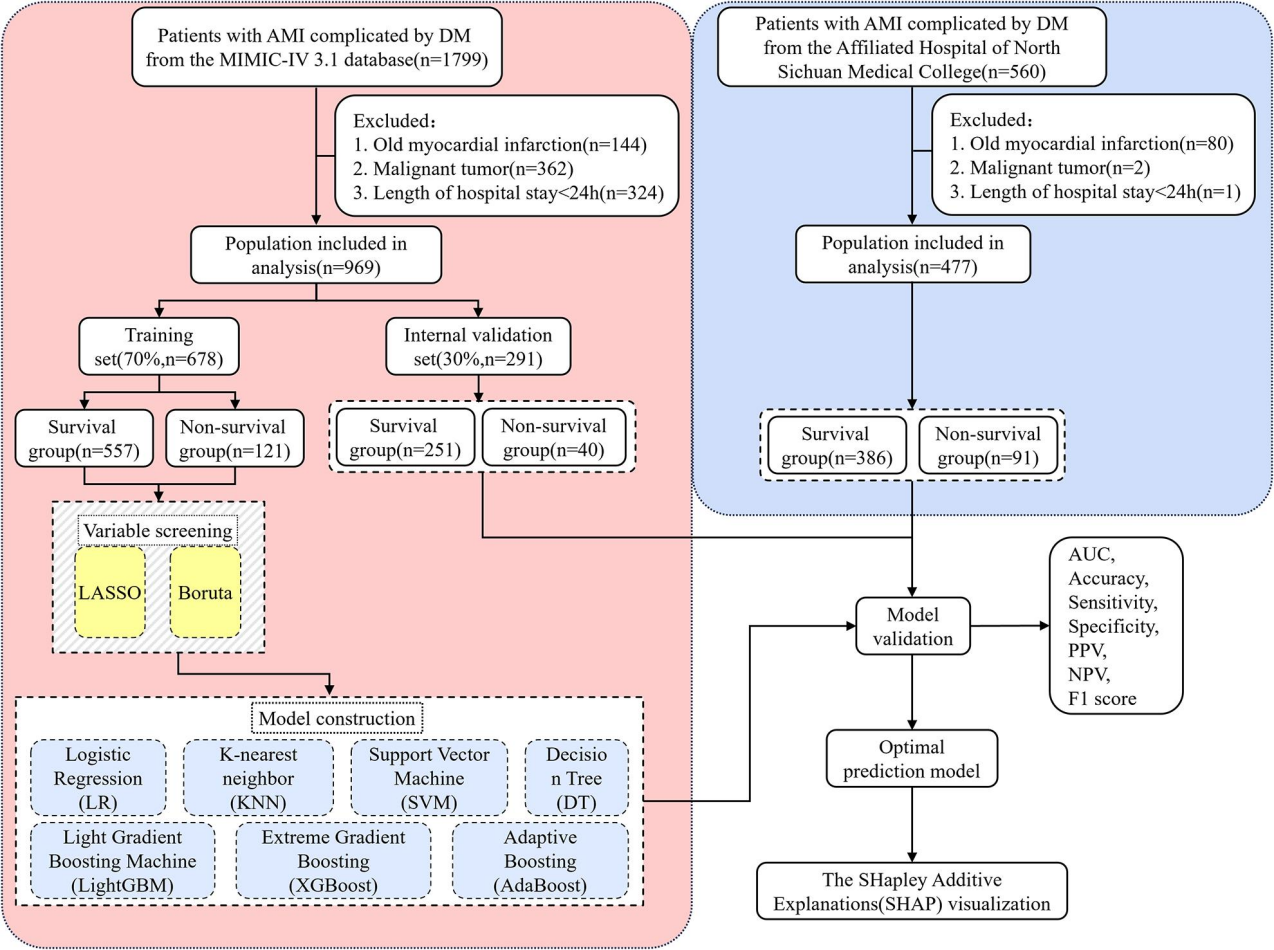
The study nflow diagram. AMI: Acute Myocardial Infarction. DM: Diabetes Mellitus. AUC: Area Under the Curve. PPV: Positive Predictive Value. NPV: Negative Predictive Value.

The study population was divided into two cohorts. The first cohort was derived from the MIMIC-IV database, for which a rigorous data cleaning procedure was applied: (1) Diagnosis confirmation: AMI and DM were jointly identified using ICD-9 and ICD-10 codes to ensure consistent and reliable diagnoses; (2) Duplicate admission processing: Duplicate hospital records were identified and excluded using three fields in the database: subject_id, hadm_id, and stay_id; (3) First admission screening: To avoid selection bias, only data from patients' first adult admission (age > 18 years) were retained. The above procedures ensured the independence and validity of the study sample. 1,799 patients with AMI complicated by DM were initially screened. After excluding 144 patients with old myocardial infarction, 362 patients with malignancy, and 324 patients whose hospital stay was less than 24 h, 969 patients were ultimately included in the study (Supplementary Table S1). This cohort was randomly divided into a training set and an internal validation set at a ratio of 7:3. The training set was used for feature selection and model construction, whereas the validation set was used for internal model validation. The second cohort consisted of 560 patients diagnosed with AMI complicated by DM at the Affiliated Hospital of North Sichuan Medical College between January 2021 and April 2025. After excluding 80 patients with old myocardial infarction, 2 patients with malignancy, and 1 patient with a hospital stay of less than 24 h, 477 patients were ultimately included. This cohort was used as an external validation set to evaluate the generalizability of the predictive models developed in this study (Figure 1).

### Data extraction and data preprocessing

Patient data were extracted from the MIMIC-IV database using PostgreSQL (version 13.7.2) and Navicat Premium (version 17). Data from 560 patients diagnosed with AMI complicated by DM between January 2021 and April 2025 were extracted from the Hospital Information System, Laboratory Information Management System, and Electronic Medical Record Systems of the Affiliated Hospital of North Sichuan Medical College. Demographic characteristics, vital signs, comorbidities, laboratory examinations, and clinical medication data (medications prescribed and administered during hospitalization) were extracted for study participants from both the MIMIC-IV database and the database of the Affiliated Hospital of North Sichuan Medical College. All study variables were based on the first measurements obtained at hospital admission. Before model training, to reduce the influence of missing data, variables with over 30% missing values in the MIMIC-IV database were excluded. The missing rates for all variables in the MIMIC-IV database are presented in Supplementary Table S2. Little's MCAR test suggested that the remaining data were not missing completely at random (MCAR) (*χ*^2^ = 2856.29, df = 2130, *P* < 0.001). Accordingly, considering the retrospective nature of this study, we assumed the data were missing at random (MAR). For variables with a missing rate below 30%, imputation was carried out using the RF algorithm (Supplementary Table S3). This algorithm exhibits strong noise resistance, is less susceptible to outliers, and has no requirements for data distribution types (30). In contrast, the patient data extracted from the database of the Affiliated Hospital of North Sichuan Medical College were complete. To address differences in scale and distribution among variables and ensure the reliability of results, continuous variables were standardized using the Z-score method to improve comparability across data indicators. The study population exhibited significant class imbalance. To improve the final predictive performance of the models, the Synthetic Minority Oversampling Technique (SMOTE, K = 5) was applied to the training set to alleviate this imbalance.

### Study outcomes

The primary outcome of this study was in-hospital all-cause mortality.

### Variable selection

After comprehensive data preprocessing, 44 variables were ultimately included in the analysis. These variables included demographic data, vital signs, clinical comorbidities, clinical medications, and laboratory findings. Demographics and vital signs include gender, age, body mass index (BMI), systolic blood pressure (SBP), diastolic blood pressure (DBP), temperature, respiratory rate (RR), and heart rate (HR). Clinical comorbidities include hypertension, heart failure, pulmonary disease, renal disease, liver disease, and peripheral vascular disease (PVD). Clinical medications include antiplatelet drugs, anticoagulants, diuretics, ACEI/ARB, and statins. All medication variables were defined as medications prescribed and administered during hospitalization. Laboratory findings include white blood cell count (WBC), neutrophil count (NEUT), lymphocyte count (LYMPH), monocyte count (MONO), red blood cell count (RBC), platelet count (PLT), hemoglobin (Hb), systemic immune-inflammation index (SII), platelet-to-lymphocyte ratio (PLR), neutrophil-to-lymphocyte ratio (NLR), prothrombin time (PT), activated partial thromboplastin time (APTT), international normalized ratio (INR), aspartate aminotransferase (AST), alanine aminotransferase (ALT), albumin, alkaline phosphatase (ALP), total bilirubin (TBiL), creatinine (Cr), blood urea nitrogen (BUN), cardiac troponin T (cTnT), blood glucose (BG), potassium, sodium, and chloride. To develop a robust and representative predictive model, two distinct methods were applied to screen for feature variables in the training cohort, ensuring the reliability and generalizability of the included variables: the Least Absolute Shrinkage and Selection Operator (LASSO) regression and the Boruta algorithm. LASSO regression was applied to analyze high-dimensional data and automatically select key variables, preventing overfitting. LASSO regression enhances model parsimony by applying a penalty coefficient, shrinking the coefficients of less important predictor variables to zero and retaining only the optimal predictors in the model (31), which enhances the model's ability to generalize to unseen data and ensures robustness. Boruta Algorithm: This method is a wrapper-based feature selection algorithm built upon the RF framework. Its core principle is to introduce randomly generated “shadow features” as a comparison benchmark. The algorithm can identify key features with statistical significance by comparing the predictive importance of original features with these random features. First, the algorithm creates shuffled copies of each original feature, designates them as shadow features, and then merges them with the original features to construct an expanded feature set. Subsequently, by iteratively training RF models, it calculates importance scores (typically represented by Z-scores) for both original and shadow features (32). A feature is retained as important and not pruned during each iteration if its calculated importance consistently surpasses the highest importance threshold observed across all shadow features; otherwise, it is deemed irrelevant and pruned. This iterative process continues until the importance status of all features stabilizes or a predetermined stopping criterion is reached, and a rigorously selected feature subset is ultimately obtained. The variables that were commonly selected by both methods were employed as the feature variables for the predictive model.

### Statistical analysis

SPSS (version 27.0), R (version 4.5.1), and Python (version 3.7.12) software were used for data analysis and processing. Continuous variables with a normal distribution were presented as mean ± standard deviation, while those without a normal distribution were presented as median (interquartile range) [M (Q1, Q3)]. The Shapiro–Wilk test was used to assess the normality of the continuous variables. Categorical variables were expressed as frequencies and percentages (%). For inter-group comparisons, Student's t-test or Mann–Whitney U test was applied to continuous variables as appropriate. Intergroup comparisons for categorical variables were performed using Pearson's chi-square test or Fisher's exact test. The R packages included “readxl”, “dplyr”, “tidyr”, “openxlsx”, “moments”, “stats”, “base”, “naniar” and “igraph”. The Python packages included “numpy”, “scipy”, “pandas”, “matplotlib”, “scikit-learn”, “imbalanced-learn”, “xgboost”, and “lightgbm”. For all tests, a two-tailed *p*-value less than 0.05 was deemed statistically significant.

### Model construction

Based on the common feature variables selected in the training set, seven machine learning methods were used to construct predictive models in this study, including Logistic Regression (LR), K-Nearest Neighbors (KNN), Support Vector Machine (SVM), Decision Tree (DT), Light Gradient Boosting Machine (LightGBM), Adaptive Boosting (AdaBoost), and Extreme Gradient Boosting (XGBoost). All models underwent hyperparameter tuning in the training set using grid search combined with 5-fold cross-validation. The hyperparameter search space and optimal hyperparameters are presented in Supplementary Table S4 and Supplementary Table S5, respectively.

### Model evaluation and interpretation

The receiver operating characteristic (ROC) analysis was performed based on the continuous probability values output by the models. For the binary classification task, the optimal cutoff threshold was determined by maximizing the Youden index only in the training set. This fixed threshold was then applied to the internal and external validation sets to avoid repeated threshold estimation in non-training datasets. The predictive performance of various ML models was assessed using multiple metrics across training, internal and external validation datasets. The metrics evaluated included the area under the ROC curve, accuracy, sensitivity, specificity, positive predictive value (PPV), negative predictive value (NPV), and F1-score. Furthermore, DeLong's test was used to assess significant differences across the seven ML models. The model with the highest external AUC and favorable overall performance was selected as the preferred predictive model. To assess the model's generalizability, subgroup analyses stratified by age (≥65 years vs. < 65 years) and sex (male vs. female) were further conducted in the internal and external validation sets to verify the predictive performance of the preferred model in different populations. To validate the robustness of the findings, prediction models were separately established using the original imbalanced data and SMOTE-corrected data, and the model performance under the two strategies was compared to evaluate the impact of class imbalance handling on the results. Optimal cutoff values for each continuous predictor were calculated in the training set using the maximum Youden index to identify thresholds for distinguishing high-risk and low-risk patients for in-hospital mortality, providing an actionable reference standard for clinical application. Decision curve analysis (DCA) was applied to assess the clinical utility of the models. Additionally, model calibration was comprehensively evaluated using calibration curves, the Hosmer-Lemeshow test, calibration intercept, calibration slope, Brier score, and observed-to-expected (O/E) ratio. Furthermore, Shapley Additive exPlanations (SHAP) was employed to conduct interpretability analysis of the model's predictions from both global and local perspectives. Model selection was performed according to a prespecified priority order: Primary: Highest AUC in the external validation set; Secondary: Favorable model calibration assessed by Hosmer–Lemeshow test, calibration curves, and quantitative calibration metrics; Third: Favorable clinical utility evaluated by DCA; Fourth: Stable and balanced performance across multiple metrics.

## Results

### Baseline characteristics

Following variable screening, 44 variables were ultimately incorporated into the analysis, covering demographic characteristics, vital signs, comorbidities, laboratory findings, and clinical medication data of the study participants. A total of 969 patients with AMI complicated by DM were identified from the MIMIC-IV database, among whom 161 experienced all-cause in-hospital death, with an in-hospital mortality rate of 16.6%. The dataset was randomly divided into a training set and an internal validation set using a 7:3 ratio. The training set comprised 678 patients (70%), with an in-hospital mortality rate of 17.8% (121/678), as shown in Table 1. The internal validation set included 291 patients (30%), with an in-hospital mortality rate of 13.7% (40/291), as presented in Supplementary Table S6. Following the application of inclusion and exclusion criteria, 477 patients diagnosed with AMI complicated by DM were selected from the database of the Affiliated Hospital of North Sichuan Medical College to serve as an independent external test set. Among them, 91 patients experienced all-cause in-hospital death, with an in-hospital mortality rate of 19.1% (91/477), as presented in Supplementary Table S7. Table 1 and Supplementary Tables S6, S7 provide a detailed comparison of the clinical characteristics between the survival and non-survival groups.

**Table 1:** Baseline characteristics between the survival group and the non-survival group in the training set.

Variables	Total (*N* = 678)	Survival (*N* = 557)	Non-survival (*N* = 121)	*P* value
Demographics				
Age(years)	70.9 (62.8−78.3)	70.4 (62.6−77.8)	73.4 (65.6−80.9)	0.011
BMI(Kg/m^2^)	29.4 (25.8−33.8)	29.3 (25.8−33.5)	30.6 (25.7−36.1)	0.175
Gender(n,%)				0.574
female	251 (37%)	203 (36.4%)	48 (39.7%)	
male	427 (63%)	354 (63.6%)	73 (60.3%)	
Vital signs				
Temperature(℃)	36.7 (36.4−36.9)	36.7 (36.4−36.9)	36.7 (36.4−37)	0.670
Heart rate(bpm)	85 (78−98)	84 (78−95)	93 (79−109)	<0.001
Respiratory rate(bpm)	19 (16−23)	18 (16−22)	21 (18−26)	<0.001
SBP(mmHg)	122 (106−135)	123 (108−135)	117 (103−133)	0.062
DBP(mmHg)	65 (56−76.4)	65 (56−76.3)	66 (55−78)	0.906
Laboratory measurements				
WBC(10^9^/L)	10.8 (8.1−14.9)	10.3 (7.9−14.2)	13.4 (9.6−18.9)	<0.001
NEUT(10^9^/L)	9.4 (6.3−13.1)	8.8 (6.1−12.3)	11.8 (8.5−17.1)	<0.001
LYMPH(10^9^/L)	1.3 (0.8−2.1)	1.4 (0.9−2.2)	1 (0.7−1.5)	<0.001
MONO(10^9^/L)	0.6 (0.4−0.9)	0.6 (0.3−0.9)	0.8 (0.5−1.2)	<0.001
RBC(10^12^/L)	3.9 (3.2−4.4)	3.9 (3.3−4.5)	3.5 (3−4.2)	<0.001
PLT(10^9^/L)	213.5 (162.2−269.8)	216 (167−272)	190 (137−265)	0.017
SII	1423.4 (694.9−2906.6)	1325.4 (664.7−2500.5)	2498.6 (1108.6−4679.4)	<0.001
PLR	156.1 (94.5−263.1)	150.5 (90.5−247.8)	198.6 (125−320.9)	<0.001
NLR	6.9 (3.7−12.7)	6.1 (3.4−11)	12 (7.1−19.3)	<0.001
Hb(g/L)	113.5 (96−130)	115 (99−132)	103 (88−118)	<0.001
PT(s)	13.3 (12−15.5)	13 (12−15.1)	14.7 (13.2−17.7)	<0.001
APTT(s)	34.3 (28.3−53.4)	34.3 (28.4−53.3)	34.2 (27.7−54.9)	0.856
INR	1.2 (1.1−1.4)	1.2 (1.1−1.4)	1.3 (1.2−1.6)	<0.001
AST(IU/L)	48 (25−121.5)	45 (24−100)	72 (36−240)	<0.001
ALT(IU/L)	32 (19−64.4)	31 (19−59)	46 (20−136)	0.001
Albumin(g/L)	34 (30.8−37)	34.8 (31−38)	30.3 (27−34)	<0.001
ALP(IU/L)	86 (67−110.6)	84 (67−106)	97 (74−130)	0.001
TBiL(mg/dL)	0.5 (0.4−0.8)	0.5 (0.4−0.8)	0.8 (0.5−1.3)	<0.001
Creatinine(mg/dL)	1.3 (0.9−2.1)	1.2 (0.9−1.9)	1.6 (1.2−3)	<0.001
BUN(mg/dL)	27 (17−46)	25 (17−43)	38 (24−64)	<0.001
cTnT(ng/mL)	0.4 (0.2−1.3)	0.4 (0.2−1.3)	0.4 (0.1−1.5)	0.858
Blood glucose(mg/dL)	189 (140.2−266)	185 (139−262)	194 (146−287)	0.280
Potassium(mmol/L)	4.3 (4−4.9)	4.3 (3.9−4.8)	4.5 (4−5.2)	0.003
Sodium(mmol/L)	138 (135−140)	138 (135−140)	137 (134−140)	0.123
Chloride(mmol/L)	101 (98−105)	101 (98−105)	101 (97−105)	0.264
Comorbidities				
Hypertension(n,%)				0.012
No	440 (64.9%)	349 (62.7%)	91 (75.2%)	
Yes	238 (35.1%)	208 (37.3%)	30 (24.8%)	
Heart failure(n,%)				0.012
No	279 (41.2%)	242 (43.4%)	37 (30.6%)	
Yes	399 (58.8%)	315 (56.6%)	84 (69.4%)	
Pulmonary disease(n,%)				0.561
No	499 (73.6%)	413 (74.1%)	86 (71.1%)	
Yes	179 (26.4%)	144 (25.9%)	35 (28.9%)	
PVD(n,%)				0.034
No	582 (85.8%)	486 (87.3%)	96 (79.3%)	
Yes	96 (14.2%)	71 (12.7%)	25 (20.7%)	
Renal disease(n,%)				0.237
No	405 (59.7%)	339 (60.9%)	66 (54.5%)	
Yes	273 (40.3%)	218 (39.1%)	55 (45.5%)	
Liver disease(n,%)				<0.001
No	628 (92.6%)	527 (94.6%)	101 (83.5%)	
Yes	50 (7.4%)	30 (5.4%)	20 (16.5%)	
Treatments				
Antiplatelet drugs(n,%)				0.014
No	148 (21.8%)	111 (19.9%)	37 (30.6%)	
Yes	530 (78.2%)	446 (80.1%)	84 (69.4%)	
Statins(n,%)				0.821
No	564 (83.2%)	462 (82.9%)	102 (84.3%)	
Yes	114 (16.8%)	95 (17.1%)	19 (15.7%)	
Anticoagulant drugs(n,%)				0.828
No	91 (13.4%)	76 (13.6%)	15 (12.4%)	
Yes	587 (86.6%)	481 (86.4%)	106 (87.6%)	
ACEI/ARB(n,%)				0.005
No	604 (89.1%)	487 (87.4%)	117 (96.7%)	
Yes	74 (10.9%)	70 (12.6%)	4 (3.3%)	
DM(n,%)				0.574
No	276 (40.7%)	230 (41.3%)	46 (38%)	
Yes	402 (59.3%)	327 (58.7%)	75 (62%)	

BMI, body mass index; SBP, systolic blood pressure; DBP, diastolic blood pressure; WBC, white blood cell count; NEUT, neutrophil count; LYMPH, lymphocyte count; MONO, monocyte count; RBC, red blood cell count; PLT, platelet count; Hb, hemoglobin; SII, systemic immune-inflammation index; PLR, platelet-to-lymphocyte ratio; NLR, neutrophil-to-lymphocyte ratio; PT, prothrombin time; APTT, activated partial thromboplastin time; INR, international normalized ratio; AST, aspartate aminotransferase; ALT, alanine aminotransferase; ALP, alkaline phosphatase; TBiL, total bilirubin; BUN, blood urea nitrogen; cTnT, cardiac troponin T; PVD, peripheral vascular disease; ACEI/ARB, Angiotensin-Converting Enzyme Inhibitor/Angiotensin II Receptor Blocker; DM, Diabetes Mellitus.

### Feature selection

**Figure 2:**
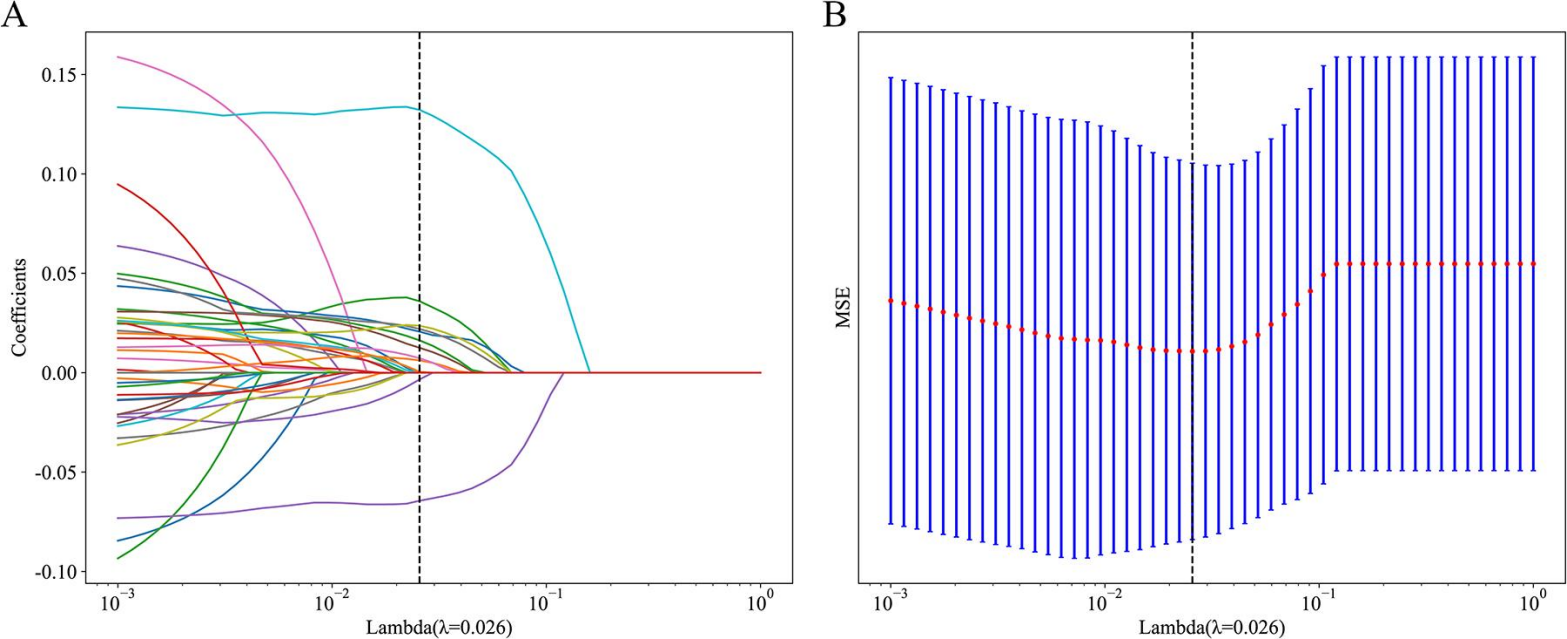
**(A)** lasso coefficient path plot. **(B)** Lasso cross-validation plot.

**Figure 3:**
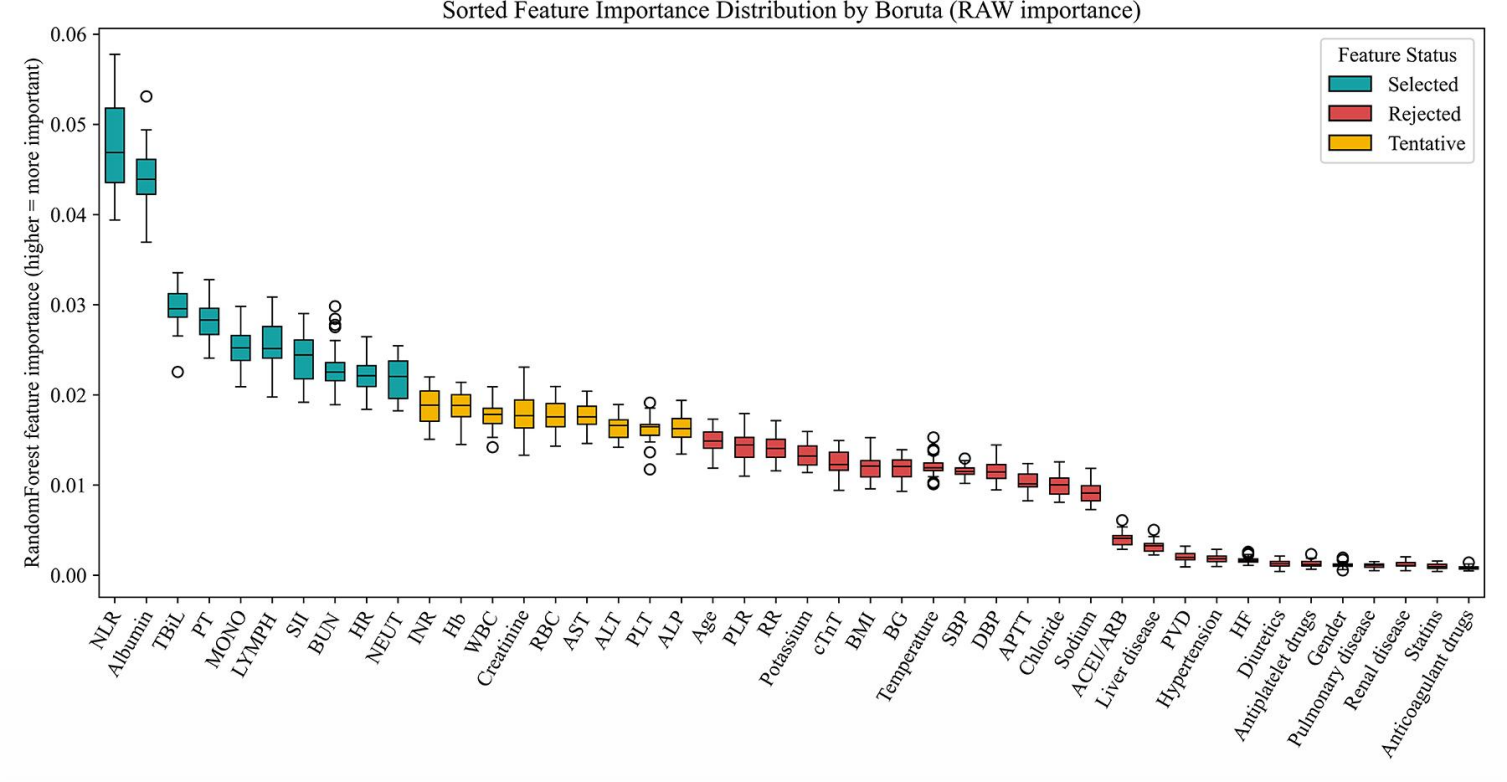
Feature selection based on the boruta algorithm. The horizontal axis represents each feature, and the vertical axis represents the Z-score of each feature. The boxplots illustrate the Z-scores of features during the selection process.

To validate the generalizability of the constructed predictive models, two different feature selection methods, namely, the LASSO regression and the Boruta algorithm, were applied during the model training phase. With in-hospital mortality as the endpoint, overlapping features identified by both methods were used as target predictors to establish the in-hospital mortality prediction model. Specifically, the LASSO regression selected 10 variables (Figure 2; Supplementary Table S8). The Boruta algorithm also identified 10 variables (Figure 3; Supplementary Table S8). These seven variables were stably selected using both feature selection approaches, indicating favorable universality and representativeness in their predictive value. Therefore, they were used as the target predictors for constructing the in-hospital mortality prediction model for patients with AMI complicated by DM (Supplementary Table S8). Supplementary Table S3 presents the relevant statistical parameters of the two feature selection methods.

### Model performance

**Figure 4:**
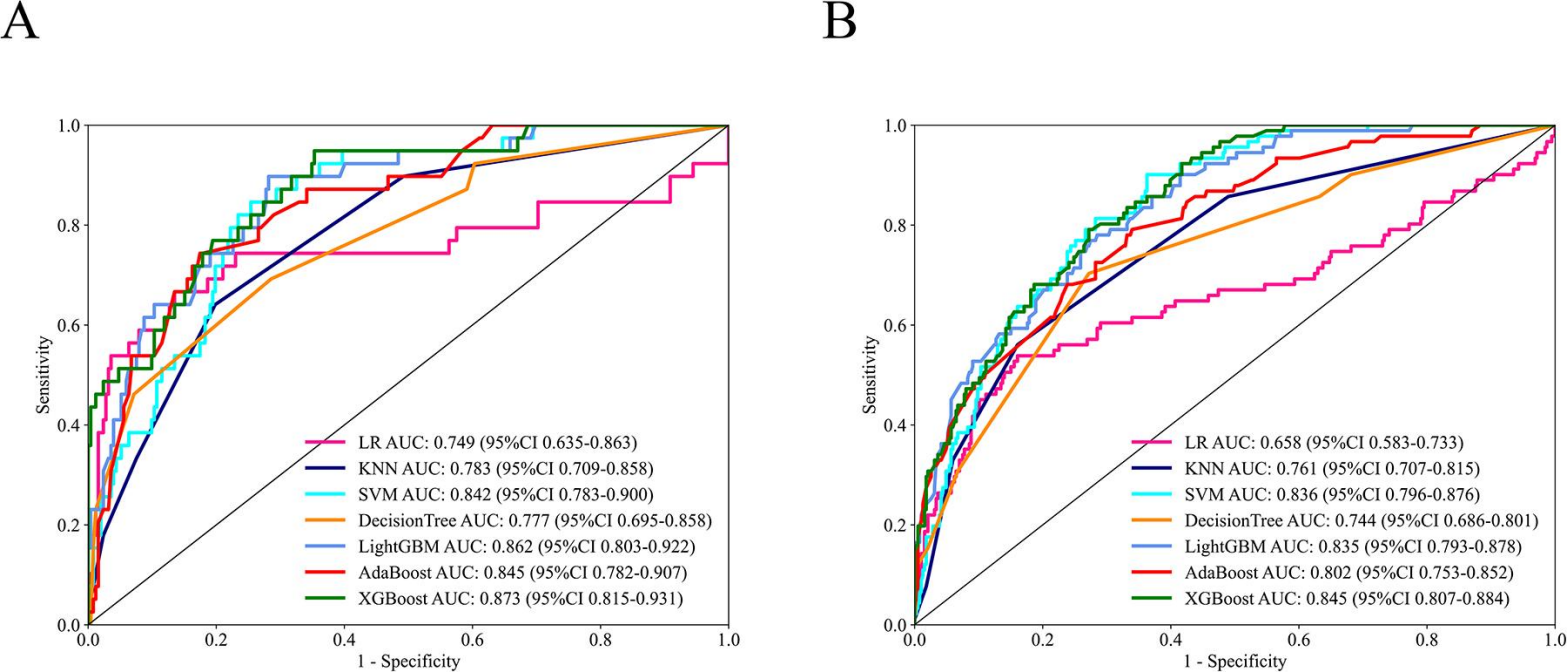
Comparison of ROC for each machine learning model. **(A)** Internal validation set. **(B)** External validation set.

**Figure 5:**
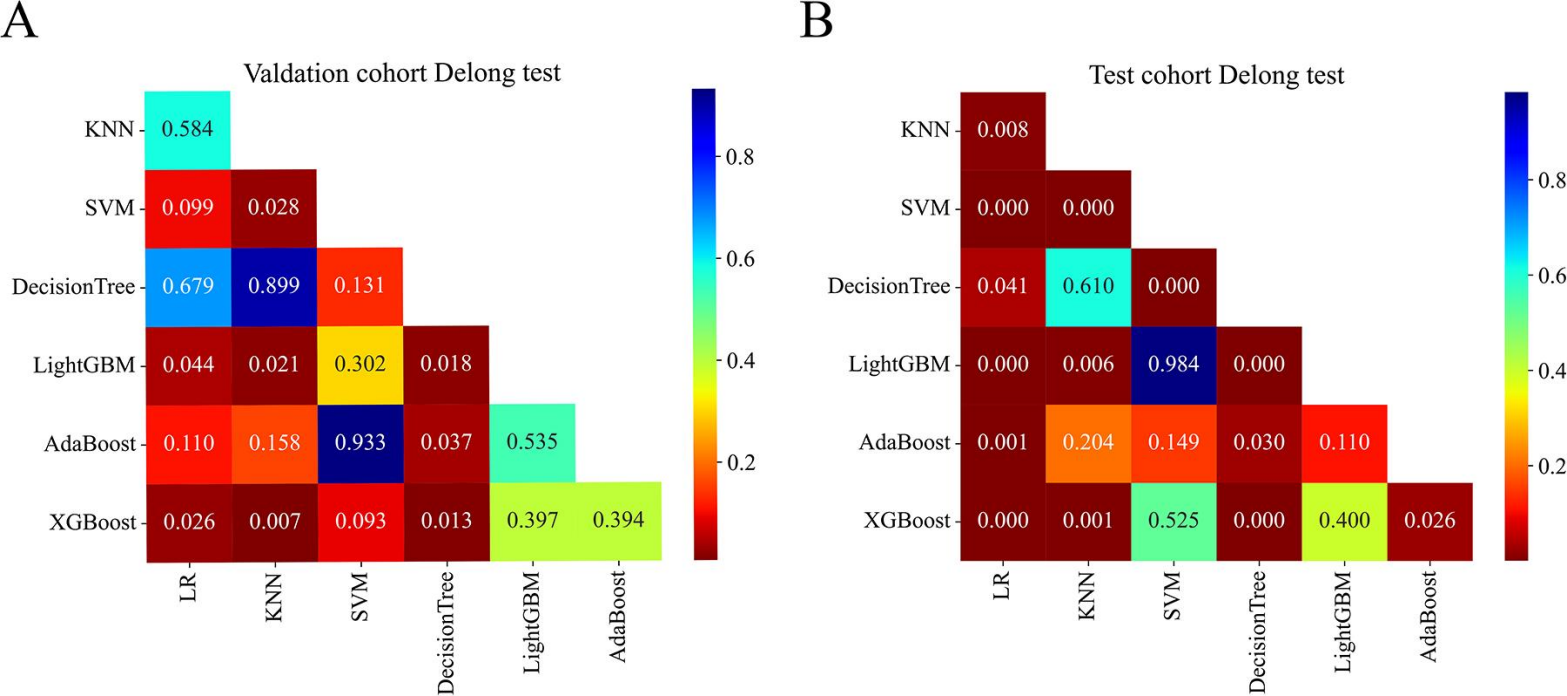
Comparison of deLong test for each machine learning model. **(A)** Internal validation set. **(B)** External validation set.

**Figure 6:**
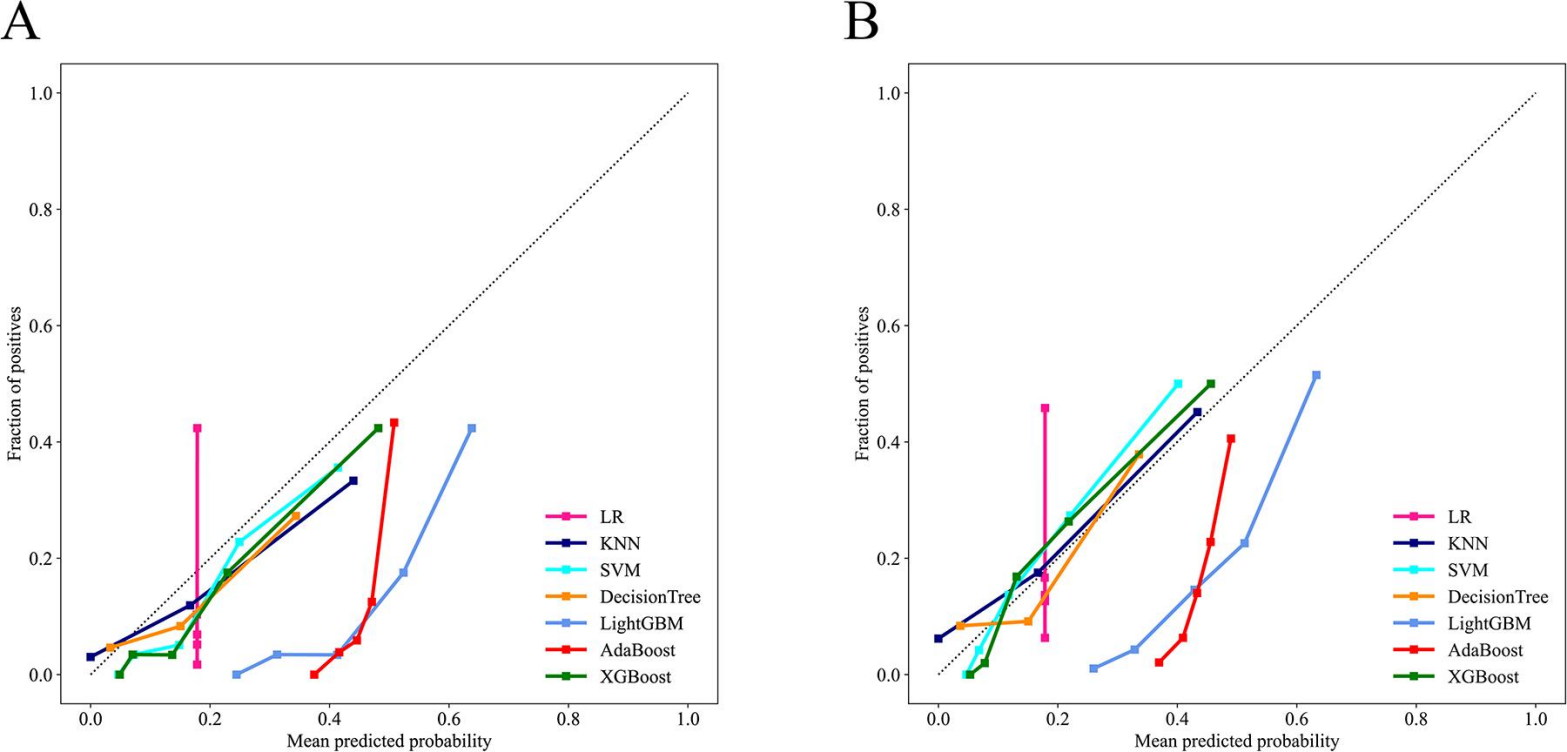
Comparison of calibration curves for each machine learning. **(A)** Internal validation set. **(B)** External validation set.

**Figure 7:**
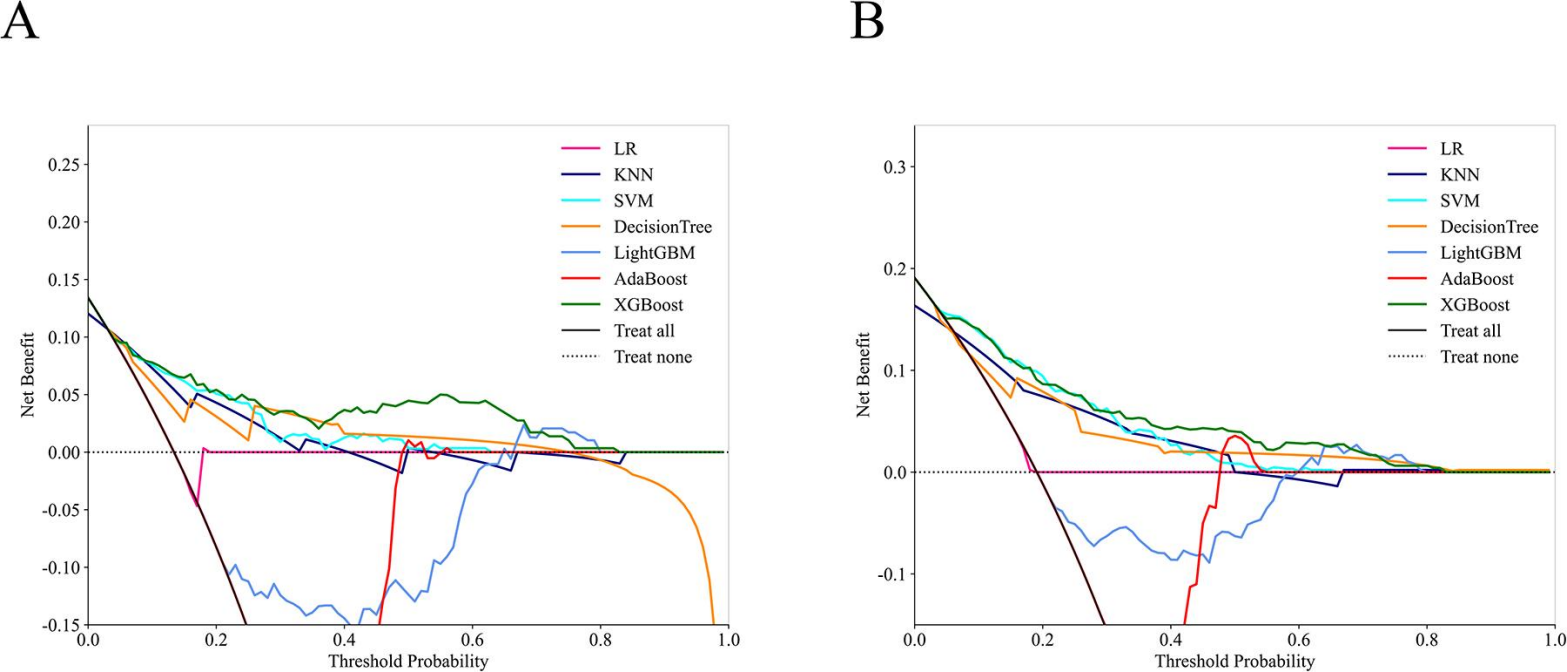
Comparison of DCA for each machine learning. **(A)** Internal validation set. **(B)** External validation set.

The AUC values and comprehensive performance metrics of the seven ML prediction models are presented in Supplementary Table S9, Figure 4, Table 2, and Table 3. All model evaluations were performed on the original, non-resampled internal and external validation cohorts without SMOTE or any resampling processing. Other evaluation metrics include accuracy, sensitivity, specificity, PPV, NPV, and F1-score. Based on a comprehensive evaluation of all indicators, the XGBoost model achieved the numerically highest AUC and favorable overall performance, and it was selected as the preferred model in the training, internal and external validation sets. In the training set, the XGBoost model achieved an AUC of 0.907 (95% CI: 0.876–0.939), with accuracy of 0.888, sensitivity of 0.752, specificity of 0.917, PPV of 0.664, NPV of 0.945, F1-score of 0.705 and cutoff of 0.281 (Supplementary Table S9). In the internal validation set, the XGBoost model yielded an AUC of 0.873 (95% CI: 0.815–0.931), with accuracy of 0.684, sensitivity of 0.923, specificity of 0.647, PPV of 0.288, NPV of 0.982, and F1-score of 0.439 (Figure 4, Table 2). In the external validation set, it achieved an AUC of 0.845 (95% CI: 0.807–0.884), outperforming the other six machine learning models, with an accuracy of 0.736, sensitivity of 0.769, specificity of 0.728, PPV of 0.400, NPV of 0.930, and F1-score of 0.526 (Figure 4, Table 3). The confusion matrices for the XGBoost model in the internal and external validation sets are presented in Supplementary Figure S1 to further illustrate its detailed classification performance. Figure 5 shows the results of DeLong's test for the internal validation and external validation sets. In the internal validation set, although the XGBoost model achieved a higher AUC than the other models, the differences were statistically significant only compared with LR, KNN, and DT (*P* < 0.05); no significant differences were observed for the remaining models (*P* > 0.05). In the external validation set, significant differences were only found when compared with LR, KNN, DT, and AdaBoost (*P* < 0.05), whereas no significant differences were found when compared with the other models (*P* > 0.05). Furthermore, the Hosmer-Lemeshow goodness-of-fit test indicated that the XGBoost model was well-calibrated, with *p* values of 0.121 and 0.089 for the internal and external validation sets, respectively, both greater than 0.05, suggesting no significant difference between the predicted probabilities and the observed outcomes (Table 4). Comprehensive quantitative calibration metrics were calculated for all models in both internal and external validation sets, including calibration intercept, calibration slope, Brier score, and O/E ratio (Supplementary Table S10). In the internal validation set, the XGBoost model showed excellent calibration with a calibration intercept of −0.03, calibration slope of 0.96, Brier score of 0.087, and O/E ratio of 0.99. In the external validation set, the XGBoost model also achieved reliable calibration with a calibration intercept of 0.02, calibration slope of 1.01, Brier score of 0.116, and O/E ratio of 1.01. The calibration curves demonstrated that the observed curve of the in-hospital mortality risk prediction model for patients with AMI complicated by DM, constructed using the XGBoost algorithm, closely followed the ideal curve with minor fluctuations around it (Figure 6). This indicated favorable agreement between the predicted probabilities and the actual observed outcomes. To facilitate bedside clinical application, optimal cutoff values for each continuous predictor were calculated in the training set using the maximum Youden index to determine high-risk thresholds. These optimal cutoffs were fixed and applied to the internal and external validation sets, with the results presented in Supplementary Table S1^1^. The clinical utility of the constructed in-hospital mortality risk model was further supported by decision curve analysis, which indicated that its application could benefit patients (Figure 7).

**Table 2:** Results of each model in the internal validation set.

Model	AUC(95%CI)	Accuracy	Sensitivity	Specificity	PPV	NPV	F1 score
LR	0.749 (0.635−0.863)	0.835	0.641	0.865	0.424	0.940	0.510
KNN	0.783 (0.709−0.858)	0.845	0.333	0.925	0.406	0.900	0.366
DT	0.777 (0.695−0.858)	0.866	0.462	0.929	0.500	0.918	0.480
XGBoost	0.873 (0.815−0.931)	0.684	0.923	0.647	0.288	0.982	0.439
LightGBM	0.862 (0.803−0.922)	0.739	0.872	0.718	0.324	0.973	0.472
AdaBoost	0.845 (0.782−0.907)	0.814	0.718	0.829	0.394	0.950	0.509
SVM	0.842 (0.783−0.900)	0.756	0.821	0.764	0.333	0.964	0.474

LR, Logistic Regression; KNN, K-Nearest Neighbors; SVM, Support Vector Machine; DT, Decision Tree; LightGBM, Light Gradient Boosting Machine; AdaBoost, Adaptive Boosting; XGBoost, eXtreme Gradient Boosting; PPV, Positive Predictive Value; NPV, Negative Predictive Value.

**Table 3:** Results of each model in the external validation set.

Model	AUC(95%CI)	Accuracy	Sensitivity	Specificity	PPV	NPV	F1 score
LR	0.658 (0.583−0.733)	0.780	0.527	0.839	0.463	0.883	0.478
KNN	0.761 (0.707−0.815)	0.824	0.330	0.940	0.566	0.856	0.417
DT	0.744 (0.686−0.801)	0.813	0.308	0.933	0.519	0.851	0.386
XGBoost	0.845 (0.807−0.884)	0.736	0.769	0.728	0.400	0.930	0.526
LightGBM	0.835 (0.793−0.878)	0.727	0.769	0.718	0.391	0.930	0.519
AdaBoost	0.802 (0.753−0.852)	0.686	0.780	0.663	0.353	0.928	0.486
SVM	0.836 (0.796−0.876)	0.686	0.890	0.637	0.367	0.961	0.519

LR, Logistic Regression; KNN, K-Nearest Neighbors; SVM, Support Vector Machine; DT, Decision Tree; LightGBM, Light Gradient Boosting Machine; AdaBoost, Adaptive Boosting; XGBoost, eXtreme Gradient Boosting; PPV, Positive Predictive Value; NPV, Negative Predictive Value.

**Table 4:** Hosmer–lemeshow test results for 7 models.

Cohort	LR	KNN	SVM	DT	LightGBM	AdaBoost	XGBoost	*P* value
Train	<0.001	0.893	<0.001	0.662	<0.001	<0.001	0.280	*P* value
Internal	0.014	0.195	0.024	0.137	<0.001	<0.001	0.121	*P* value
External	<0.001	0.033	<0.001	0.568	<0.001	<0.001	0.089	*P* value

LR, Logistic Regression; KNN, K-Nearest Neighbors; SVM, Support Vector Machine; DT, Decision Tree; LightGBM, Light Gradient Boosting Machine; AdaBoost, Adaptive Boosting; XGBoost, eXtreme Gradient Boosting.

### Subgroup analysis

To further evaluate the stability and applicability of the XGBoost prediction model across different populations, stratified subgroup analyses were performed based on age (<65 years vs. ≥ 65 years) and sex (male vs. female). Model performance was assessed separately in the internal and external validation sets. The results are presented in Supplementary Table S12. The model achieved AUC values ranging from 0.816 to 0.869 across all subgroups, indicating good discriminative ability. The NPV was ≥0.93 in all subgroups, indicating that the model is highly effective in ruling out low-risk individuals. However, in the external validation set, the PPV was only 0.386 for the subgroup aged <65 years. Furthermore, the PPV was below 0.45 for all sex subgroups. These findings indicate that the model has a limited ability to accurately predict in-hospital mortality.

### Comparison of results based on SMOTE oversampling

To evaluate the impact of class imbalance on model performance, prediction models were constructed separately using the original data without SMOTE processing and the data adjusted by SMOTE oversampling. Comparisons were performed in the internal and external validation sets. XGBoost remained the preferred model under both conditions. In the internal validation set, the model based on raw data achieved an AUC of 0.876 (95% CI: 0.818–0.933), while the SMOTE-adjusted model yielded an AUC of 0.873 (95% CI: 0.815–0.931), showing high consistency. After SMOTE processing, sensitivity increased from 0.897 to 0.923, specificity decreased from 0.726 to 0.647, PPV decreased from 0.337 to 0.288, and NPV remained high (0.979 vs. 0.982) (Supplementary Table S1^3^). In the external validation set, the raw data model had an AUC of 0.846 (95% CI: 0.808–0.885), and the SMOTE-corrected model had an AUC of 0.845 (95% CI: 0.807–0.884), with virtually no difference. Sensitivity decreased slightly from 0.791 to 0.769, specificity remained unchanged at 0.728, accuracy was similar (0.740 vs. 0.736), PPV was slightly reduced from 0.407 to 0.400, NPV remained stable (0.937 vs. 0.930), and F1 score showed no obvious change (0.537 vs. 0.526) (Supplementary Table S1^3^).

### Model interpretation

**Figure 8:**
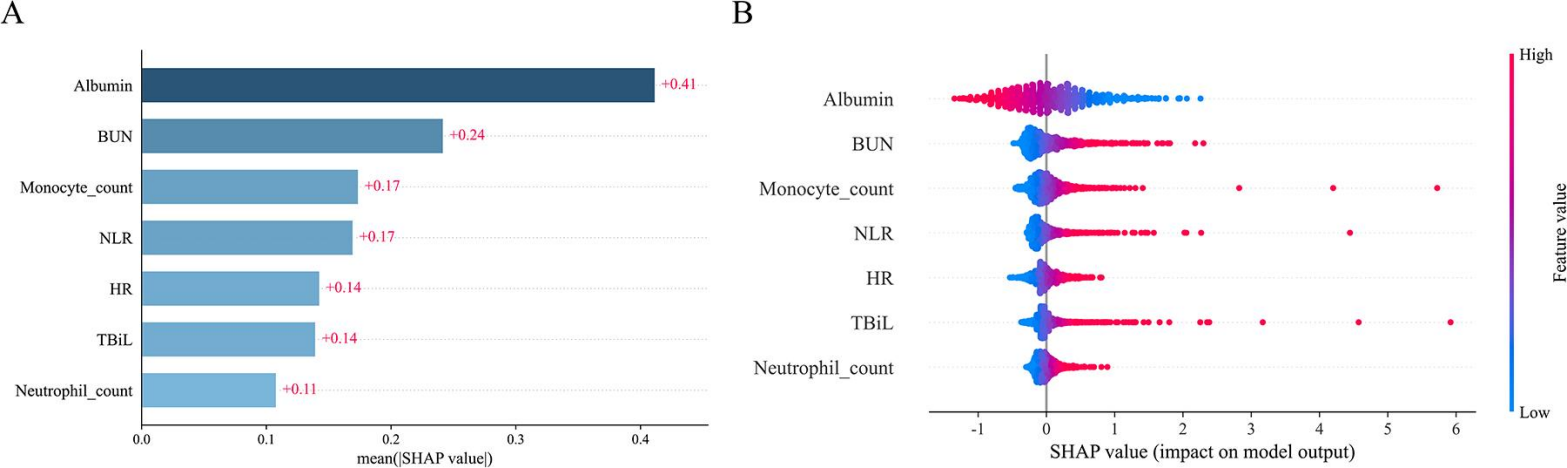
Statistical graph showing data from the SHAP analysis. **(A)** Variable importance order plot based on the SHAP analysis. **(B)** Statistical graph of the variable contribution based on the SHAP analysis.

**Figure 9:**
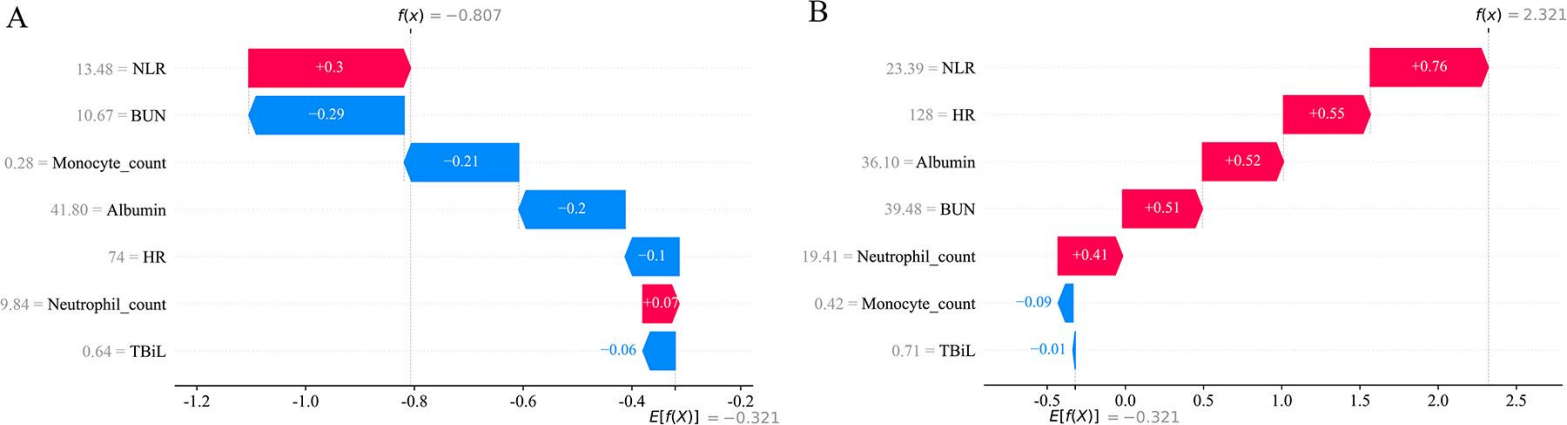
Example of risk factor analysis for patients in the external validation set. SHAP waterfall plot. **(A)** survival. **(B)** non-survival.

**Figure 10:**
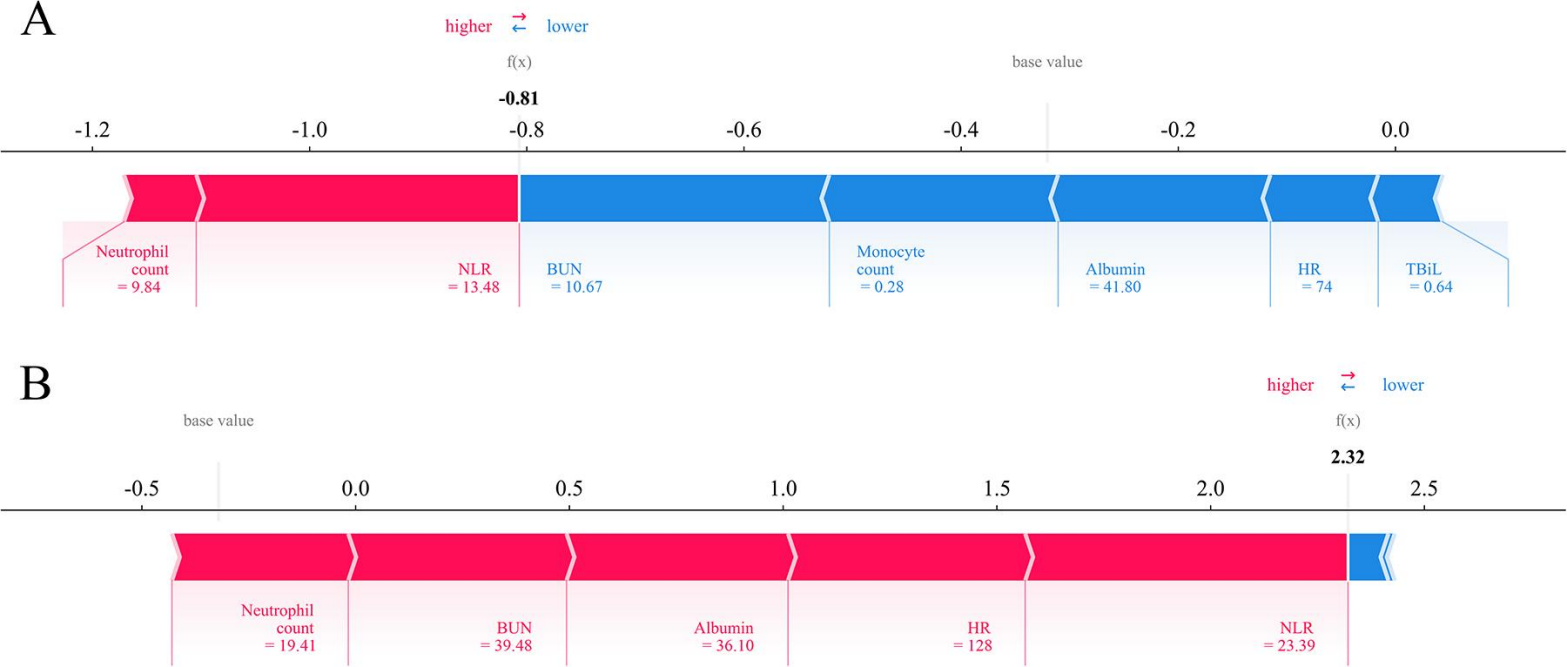
Example of risk factor analysis for patients in the external validation set. SHAP force plot. **(A)** survival. **(B)** non-survival.

To further investigate the characteristic variables associated with in-hospital mortality risk, the SHAP method was used to enhance the interpretability of the XGBoost model. The SHAP values were used to quantify the contribution of each predictor to the risk of in-hospital mortality in patients with AMI complicated by diabetes, thereby providing a clear interpretable basis for model predictions. Figure 8A shows the feature importance ranking (mean absolute SHAP values) in descending order. Serum albumin was identified as the most important predictor, followed by BUN, MONO, NLR, HR, TBiL, and NEUT. Figure 8B illustrates the distribution of SHAP values and their directional effects. The horizontal axis represents the SHAP value, with larger absolute values indicating a greater impact on in-hospital mortality. Points are color-coded by feature value: red indicates higher values, and blue indicates lower values. A positive SHAP value indicates a positive contribution (increased mortality risk), whereas a negative SHAP value indicates a negative contribution (decreased mortality risk). For instance, the risk of in-hospital mortality declined when serum albumin levels rose, indicating that serum albumin serves as a protective factor against mortality. Conversely, elevated BUN, MONO, NLR, HR, TBiL, and NEUT levels were linked to a heightened risk of in-hospital mortality in patients with AMI complicated by DM. This illustrates the global interpretability that the SHAP method offers regarding the impact of features on the predictive model. Moreover, SHAP analysis allows for the individualized interpretation of prediction results for patients with different clinical characteristics. This study selected two patients from the external validation set as examples: one who survived and one who died. For the surviving patient, who had a HR of 74 beats per minute, NEUT of 9.84 × 10⁹/L, MONO of 0.28 × 10⁹/L, NLR of 13.48, albumin level of 41.8 g/L, TBiL of 0.64 mg/dL, and BUN of 10.67 mg/dL, SHAP analysis revealed that NLR and NEUT increased the SHAP value by 0.3 and 0.07, respectively, indicating that elevated levels of these two factors increase the risk of in-hospital mortality. In contrast, the SHAP values for BUN, MONO, albumin, HR, and TBiL decreased by 0.29, 0.21, 0.2, 0.1, and 0.06, respectively, indicating a potential protective effect on patient prognosis (Figure 9A). Figure 9B shows the SHAP analysis for the deceased patient. Furthermore, this study employed SHAP force plots to illustrate the contributing factors for these two patients (Figure 10). The red arrows pointing to the right indicate that the feature value is higher than the population average, with a positive SHAP value that pushes the prediction higher, i.e., increasing the risk of mortality. Conversely, the blue arrows pointing to the left signify that the feature value is below the population average, accompanied by a negative SHAP value that drives the prediction downward, thereby decreasing the risk of mortality. Thus, the SHAP method provides interpretability for the predictive model at both the global and individual levels.

## Discussion

Cardiovascular disease has emerged as the leading cause of death worldwide. Among its most critical clinical manifestations, AMI is characterized by sudden onset, rapid progression, high incidence, and elevated mortality rates (8). AMI significantly impairs patients' daily living abilities and directly threatens their lives, posing a substantial challenge to healthcare systems worldwide. DM is a prevalent clinical condition characterized by long-term metabolic dysregulation that results in chronic progressive damage and dysfunction in multiple organ systems, including the vasculature, nerves, heart, kidneys, and eyes-due to persistent hyperglycemia. The high prevalence of DM has established it as another major global public health challenge (33, 34). As an independent risk factor for AMI, DM is associated with coronary atherosclerosis and a hypercoagulable state. Approximately 26%–50% of patients with DM have concomitant coronary heart disease, and this pathological basis significantly increases the risk of AMI, posing a serious threat to patients' health (35). For AMI patients complicated by DM, the clinical presentation often includes more comorbidities and more complex coronary anatomy. On the one hand, persistent hyperglycemia and insulin resistance contribute to the accumulation of advanced glycation end-products (AGEs), which in turn activate inflammatory pathways and impair vascular endothelial repair, exacerbating the progression of chronic complications in DM. This, in turn, increases the likelihood of requiring repeat coronary revascularization (36). On the other hand, DM patients often exhibit heightened platelet reactivity and an inadequate response to conventional antiplatelet therapy, which significantly elevates the risk of in-stent thrombosis following coronary stent implantation (37). These complex factors lead to a higher risk of in-hospital mortality in patients with AMI and diabetes than in patients without diabetes. Therefore, effectively identifying high-risk individuals is critical for preventing in-hospital mortality among patients with AMI complicated by DM. To date, substantial research has been conducted on developing predictive models for mortality risk in AMI patients (38). However, predictive models specifically targeting in-hospital mortality risk in patients with AMI complicated by DM remain scarce. Therefore, the establishment of such a model could provide substantial support for clinicians in clinical decision-making and patient management.

In recent years, with the rapid advancement of artificial intelligence (AI), it has garnered significant attention across various industries. Within the healthcare sector, machine learning, a branch of AI, has demonstrated remarkable efficacy in analyzing medical data. This capability is attributed to the ability of machine learning to process massive, nonlinear, and high-dimensional medical data accurately and efficiently, as well as its flexibility regarding parameter assumptions. It has been widely applied in the medical domain, offering new perspectives and tools for clinical decision-making. Wu et al. demonstrated that, compared with traditional predictive models, risk assessment models constructed using machine learning algorithms (such as XGBoost) achieved higher accuracy in predicting the risk of in-hospital cardiac arrest in patients with acute coronary syndrome (39). Similarly, Huang et al. indicated that models developed using machine learning algorithms enhanced the predictive accuracy for acute kidney injury risk following percutaneous coronary intervention (PCI) compared with conventional models (23). Furthermore, Gong et al. employed machine learning methods to develop a predictive model for in-hospital mortality (40). Therefore, this study employed machine learning methods to construct a prediction model for in-hospital mortality risk in patients with AMI complicated by DM.

This study used a dual-center database to develop and validate the model. The MIMIC-IV database data were chosen as the foundational dataset for model training and internal validation. An independent dataset of Asian patients from the Affiliated Hospital of North Sichuan Medical College was employed as an external validation set to evaluate the model's applicability and transferability across different populations. Two feature selection approaches were utilized to improve the representativeness and generalizability of predictor selection while circumventing the limitations inherent in a single selection method. Ultimately, the variables consistently selected by both methods were designated as the feature set for predicting the risk of in-hospital mortality among patients with AMI complicated by DM. From a methodological standpoint, seven ML algorithms were employed to develop the predictive models. In evaluating model performance, a comprehensive approach was taken, incorporating various metrics, such as the AUC, accuracy, sensitivity, specificity, PPV, NPV, and F1-score. Upon comparison, the XGBoost model achieved the numerically highest external AUC and favorable overall performance. Notably, LightGBM and SVM also exhibited comparable discriminative performance. The XGBoost model also demonstrated superior performance with AUC values of 0.873 and 0.845 in internal and external validation sets, respectively, demonstrating its favorable generalizability in our study cohorts. DCA indicated that this predictive model might benefit patients. The calibration curves, combined with the quantitative calibration metrics, collectively demonstrated favorable agreement between predicted probabilities and observed outcomes. The model achieved an AUC of ≥0.816 across all subgroup analyses, indicating favorable discriminative ability. Furthermore, the SHAP method was utilized to offer interpretability for the XGBoost model, enabling the quantification and visualization of each feature variable's contribution to the prediction results at both global and individual levels.

This study found that SMOTE oversampling slightly increased sensitivity in the internal validation set (0.897→0.923) but reduced specificity (0.726→0.647) and PPV (0.337→0.288). In the external validation set, SMOTE led to a slight reduction in sensitivity (0.791→0.769), with unchanged specificity (0.728) and nearly identical PPV (0.407→0.400). The AUC remained highly consistent across both cohorts, indicating that the data balancing strategy did not significantly alter discriminative performance, supporting the robustness of our results. These findings confirm that applying SMOTE only in the training set did not substantially improve PPV in the validation cohorts, which is consistent with the low prevalence of in-hospital mortality events. Although the XGBoost model achieved the highest AUC in both validation cohorts, its PPV was modest due to the low event rate. Accordingly, this model exhibits excellent performance for ruling out low-risk patients (high NPV) and should be utilized as an auxiliary risk-stratification tool rather than a definitive predictive test in clinical practice.

The findings of this study suggest that NEUT, MONO, and NLR are independent risk factors for in-hospital mortality in patients with AMI complicated by DM. These indicators reflect the systemic inflammatory status. In the present study, regardless of the dataset (training set, internal validation set, or external validation set), patients in the non-survival group exhibited higher levels of MONO, NEUT, and NLR than those in the survival group, suggesting a more intense state of stress and inflammatory response in the non-survival group. As key inflammatory cells, neutrophils, lymphocytes, and monocytes play critical roles in the inflammatory response and pathological progression of AMI and serve as important biological indicators for predicting adverse cardiovascular events in patients with AMI (41). Inflammation plays a crucial role in the initiation and progression of cardiovascular disease, as inflammatory factors directly contribute to the formation of atherosclerotic plaque, thrombosis, plaque rupture, and myocardial cell injury (42). Neutrophils make up the largest proportion of leukocytes and are crucial effector cells in acute inflammatory responses. During inflammation, activated neutrophils, on one hand, release substantial amounts of reactive oxygen species and, on the other hand, form neutrophil extracellular traps (NETs). Both products promote thrombosis and accelerate coagulation processes, thereby advancing atherosclerosis, worsening myocardial injury, and causing microvascular obstruction (43). Previous studies have demonstrated that, in the context of AMI, NEUT at admission and on the first day of hospitalization are significantly positively correlated with myocardial infarct size (44). Higher NEUT are correlated with more severe myocardial injury and an increased risk of mortality in patients with AMI. Monocytes, another type of inflammatory cell, are driven by chemokines and cytokines during the inflammatory response, accelerating their aggregation to the vascular endothelium and differentiation into macrophages, which then transform into foam cells. This process represents another important pathological mechanism driving the initiation and progression of atherosclerosis, thereby aggravating myocardial injury and increasing mortality risk (45). Elevated monocyte levels are associated with a higher risk of mortality. NLR, a novel inflammatory indicator that integrates neutrophil and lymphocyte counts, has been increasingly recognized for its role in reflecting the dynamic balance of the immune system and is utilized in the diagnosis, assessment of severity, and prognosis of various diseases, reflecting both increased neutrophil counts and decreased lymphocyte counts. A decrease in lymphocyte count indicates a weakened immune regulatory function, thereby reducing the body's ability to modulate the inflammatory response. This can further lead to persistent neutrophil activation and accelerated thrombin generation, thereby worsening myocardial cell injury and increasing mortality risk (46). Elevated NLR levels in patients with AMI are associated with more intense inflammatory responses and poorer prognosis (47). Previous studies have also substantiated the link between elevated NLR levels and an increased risk of mortality. For example, research by Chen et al. (48) demonstrated that NLR is significantly associated with an increased risk of in-hospital mortality in elderly patients with AMI and can serve as an independent risk factor for predicting in-hospital death in this population. Additionally, Wang et al. (49) indicated that NLR is an independent predictor of in-hospital mortality in patients with AMI.

Heart rate is one of the most frequently employed vital signs in clinical practice, as it can swiftly reflect a patient's fundamental physiological status and aid in evaluating disease severity. In this study, patients in the non-survival group demonstrated higher HR levels than those in the survival group, suggesting that patients with markedly elevated heart rates face an increased risk of in-hospital mortality. This finding is consistent with previous studies (50, 51), which have demonstrated that compensatory tachycardia following AMI leads to shortened diastole and increased myocardial oxygen consumption, thereby worsening myocardial injury and enlarging infarct size. This may subsequently lead to severe clinical outcomes, such as cardiac dysfunction, malignant arrhythmias, and even cardiovascular death. Furthermore, Xie et al. (9) also identified HR as an independent risk factor for in-hospital mortality in patients with AMI.

Albumin is a crucial indicator of a patient's nutritional status. Serum albumin levels have been established as an independent prognostic biomarker in the clinical prognostic assessment of AMI, with decreased levels significantly associated with disease severity and adverse clinical outcomes (52). The close relationship between albumin levels and adverse cardiovascular outcomes can likely be attributed to an increased inflammatory burden and diminished antioxidant capacity and the presence of a hypercoagulable state. Furthermore, as an antioxidant, albumin can mitigate the adverse effects of oxidative stress on endothelial function by neutralizing reactive oxygen species (ROS), thereby reducing the risk of dysfunction (53). Low albumin levels frequently suggest malnutrition in patients. Patients often encounter an imbalance in energy metabolism when malnutrition arises, mainly characterized by inhibited glycolysis and enhanced fatty acid oxidation pathways. This exacerbates the energy deficit in damaged myocardial cells and may cause additional harm to the organism due to excessive production of reactive oxygen species and inflammatory factors (54). Proteins are essential for both the structural integrity and functional maintenance of cardiomyocytes. When protein levels are low, cardiomyocyte regeneration is impeded, and the time required for repair of damaged myocardium is correspondingly prolonged (55), which significantly impacts patient prognosis. Moreover, malnutrition can lead to immune cell dysfunction, including impaired macrophage and T lymphocyte phagocytic function. This reduces the body's efficiency in clearing cellular debris and inflammatory mediators from the myocardial infarction area, thereby prolonging the inflammatory response. Consequently, this triggers oxidative stress, further exacerbating myocardial damage and hindering the repair of injured myocardium (55). Oduncu et al. (56) demonstrated that patients with hypoalbuminemia not only face higher short-term risks of in-hospital mortality, heart failure, and major bleeding but also have a poorer long-term prognosis. Previous research has also indicated that the albumin level at admission is an independent predictor of long-term all-cause mortality in patients with AMI, with lower levels associated with higher mortality risk (57).

Blood urea nitrogen is the primary end product of protein metabolism in the body. Its clearance relies on the kidneys' excretory function, making it a classic indicator for assessing renal function (58). Elevated BUN levels primarily reflect reduced renal excretory capacity, with the underlying mechanism potentially involving renal hypoperfusion due to hemodynamic changes, which subsequently activates the renal sympathetic nervous system and the renin-angiotensin-aldosterone system pathway, creating a vicious cycle (59). In recent years, the clinical significance of BUN has expanded from renal function assessment to disease prognosis prediction. It has been validated as an effective predictor of adverse outcomes in various diseases, such as acute ischemic stroke, acute decompensated or chronic heart failure, and acute pulmonary embolism (60). Studies have similarly confirmed its prognostic value within the realm of AMI. For instance, a prospective study demonstrated that elevated BUN levels significantly predict the risk of in-hospital mortality in patients with AMI (61). Yuan et al. also showed an association between elevated BUN and increased in-hospital mortality in patients with AMI (62). In line with previous research, the current study reaffirms that elevated admission BUN levels are independently associated with increased in-hospital mortality risk among patients diagnosed with AMI. Statistical analysis revealed that patients in the non-survival group had significantly higher BUN levels than those in the survival group.

Total bilirubin, the sum of direct and indirect bilirubin, is the bloodstream's terminal product of heme catabolism. Concurrently, it functions as an endogenous antioxidant with significant physiological roles (63), exerting cytoprotective effects through multiple mechanisms: first, inhibiting oxidative modification of low-density lipoprotein cholesterol (LDL-C); second, mediating anti-inflammatory signaling pathways to downregulate inflammatory responses; and third, directly scavenging reactive oxygen species. These actions collectively antagonize the cytotoxicity induced by free heme released from extracellular hemoglobin (64). For instance, Lan et al. (65) demonstrated that elevated TBiL levels are a significant positive predictor of improved prognosis in patients with atherosclerotic cardiovascular disease. However, the present study found that TBiL levels were higher in the non-survival group than in the survival group among patients with AMI, indicating that a higher TBiL level was associated with an increased risk of in-hospital mortality. This finding appears to contradict the traditional understanding of the antioxidant and anti-inflammatory protective effects of bilirubin. Nevertheless, this seemingly contradictory phenomenon can still be reasonably explained. Studies have demonstrated that elevated TBiL levels are significantly correlated with adverse vascular pathological features during the acute phase of AMI, including a higher thrombus burden, exacerbated coronary atherosclerotic burden, and the coronary no-reflow phenomenon on angiography (63). These pathological processes intensify myocardial injury and limit the effectiveness of revascularization, thereby directly increasing the risk of mortality. Additionally, in patients with AMI, severe myocardial injury can lead to heart failure, which subsequently causes hepatic congestion and decreased liver perfusion, resulting in elevated TBiL levels. This may indirectly reflect cardiac dysfunction and serve as a marker of poor prognosis (66). Studies have consistently shown that increased TBiL levels are significant predictors of poor outcomes in patients experiencing AMI. For example, Chung et al. (67) identified high TBiL levels as a predictor of in-hospital adverse events and cardiac death in patients with AMI. Acet et al. (68) and Gul et al. (69) also found that elevated bilirubin levels were independent predictors of in-hospital mortality in patients with AMI. Therefore, given the dual role of TBiL described above, more prospective and multicenter studies are needed to further clarify the dynamic changes of total bilirubin in AMI and its exact clinical predictive value.

This study has several strengths. First, an in-hospital mortality risk prediction model was developed for a specific population of patients with AMI complicated by DM and its generalizability and transportability were evaluated using both internal and external validation. Second, the overlapping variables, which were selected by two feature selection methods-the LASSO regression and the Boruta algorithm-were employed as predictors. This approach ensured the robustness and generalizability of the included variables. Third, various ML algorithms were applied to construct prediction models, and their performance was rigorously evaluated using multiple metrics via internal and external validation to determine the preferred model, thus achieving a meaningful integration of medicine and AI. Fourth, the SHAP method was used to provide visual interpretations of the model's predictions, and all included predictors are readily accessible in clinical practice. Although this facilitates risk stratification of patients, the model only provides auxiliary reference and cannot serve as an independent basis for clinical decision-making. Fifth, although the development cohort was US ICU-based and the validation cohort was Chinese hospital-based, the core pathophysiology of AMI complicated by diabetes is consistent across populations. The seven predictive features are routinely measured and biologically universal. The external validation (AUC=0.845) and consistent subgroup performance provide initial support for the potential transportability of this model only to clinical settings reasonably similar to our study cohorts, including critically ill ICU populations and general cardiology inpatients with AMI complicated by DM. Caution must be exercised when applying this model to broader or dissimilar clinical practice.

## Limitations

This study has several limitations. First, the analysis was restricted by the inherent variables within the public database due to its retrospective design, leading to a relatively limited set of included variables. The key parameters, including electrocardiogram and echocardiography findings, were unavailable. Future research should incorporate large-scale, multicenter, prospective studies to ensure a more comprehensive set of variables, thereby enhancing the representativeness and applicability of the results. Second, during the data processing phase, all laboratory measurements extracted from the database were directly utilized, without employing statistical methods such as winsorizing or trimming to account for outliers. This methodology was designed to maintain the dataset's integrity by recognizing that outliers could accurately represent patients' pathophysiological conditions. However, this method also entails certain risks, as rare cases of measurement errors or data entry anomalies could potentially influence the model estimates. Third, although random forest imputation was used to handle missing data, missing-data processing still introduces potential uncertainty into the results. Little's MCAR test confirmed that data were not missing completely at random, and our MAR assumption for imputation may have slightly influenced variable distributions and the stability of feature selection. Serum albumin, the most important predictor in the SHAP analysis, had a 28.9% missing rate before imputation, which may have a subtle effect on its feature importance ranking. In addition, missing-data imputation may marginally affect model fitting and predictive performance, especially sensitivity and PPV. However, all imputed variables had missing rates controlled within 30%, and the RF algorithm is highly robust; thus, such impacts are limited and acceptable. In future studies, large-sample prospective research with complete data collection or sensitivity analyses using multiple imputation methods could be performed to further reduce uncertainty and enhance model stability. Fourth, the development cohort was derived from an ICU-based database (MIMIC-IV), whereas the external validation cohort was obtained from a general cardiology ward in a single Chinese center. These two settings differ substantially in case mix, disease severity, care intensity, and health care context. Furthermore, the single-center external validation limits geographic and demographic representativeness. Collectively, these factors restrict the model's transportability only to clinical settings reasonably similar to our study cohorts, and broad generalization to unselected routine clinical practice is not supported. Therefore, further validation in large-sample, multicenter, and prospective studies is still required before wider application. Fifth, this study's primary endpoint was in-hospital all-cause mortality, without further distinction between cardiovascular and non-cardiovascular death. The predictive specificity of the model for cardiovascular death could not be determined, which may somewhat compromise the precise assessment of cardiac-related mortality risk. Future studies may adopt cardiovascular death as an independent endpoint to further improve the specificity and clinical applicability of the model. Sixth, several key clinical variables with substantial impacts on in-hospital mortality were not included in this study, including AMI subtypes (STEMI/NSTEMI), diabetes type, duration of diabetes, glycated hemoglobin (HbA1c), reperfusion strategies (PCI, thrombolysis, CABG), Killip classification, number of diseased coronary vessels, peak myocardial enzyme levels, blood glucose variability, occurrence of hypoglycemia, and insulin regimens. These indicators are closely associated with disease severity and mortality risk in patients with AMI complicated by DM; they were excluded because either they lacked structured records in the MIMIC-IV database (e.g., number of diseased vessels, Killip class) or their extraction from the database would require extensive processing of incomplete or unstructured data (e.g., HbA1c, glucose variability, insulin regimens, reperfusion strategies), which somewhat reduces the completeness and comprehensive predictive ability of the model. Accordingly, this model should be regarded as an exploratory and auxiliary risk stratification tool for early identification of high-risk individuals, rather than an independent or decisive basis for clinical decision-making. Future studies should incorporate more comprehensive key clinical indicators to further optimize the model. Seventh, although the model maintained favorable discriminatory performance in age and sex subgroups (all AUC > 0.816), the PPV was generally low, indicating limited prediction accuracy and a relatively high false-positive rate for in-hospital mortality in patients with AMI complicated by DM. Therefore, this model is more suitable as a clinical screening tool to identify low-risk individuals, whereas “high-risk” predictions should be interpreted comprehensively in combination with other clinical indicators. Future research should aim for prospective validation in multicenter, large-sample cohorts and include more specific variables associated with mortality risk to further improve the positive predictive performance of the model. Eighth, and importantly, our model development relied on a single random 7:3 train-internal validation split, with 5-fold cross-validation solely used for hyperparameter tuning within the training set. This validation strategy is methodologically less robust than repeated resampling, nested cross-validation, or bootstrap-based optimism correction. Because model performance estimates from a single split can be subject to high variance, this fundamentally limits the stability and reproducibility of our current findings. Therefore, the reported model performance should be interpreted with this inherent uncertainty in mind. Future studies with more rigorous and comprehensive validation frameworks are essential to verify and optimize the model's performance.

## Conclusions

This study developed predictive models for in-hospital mortality risk among patients with AMI complicated by DM using seven ML algorithms based on dual-center data. After a comprehensive performance evaluation using multiple metrics, the XGBoost-based model achieved favorable overall performance in our specific single-split validation and was selected as the preferred model. Furthermore, the SHAP method was applied to visually interpret the model, improving its interpretability and clinical credibility by revealing the contribution of individual features to prediction outcomes. This model can provide auxiliary reference for the early risk identification in patients with AMI complicated by DM in similar populations, but it is only an exploratory and auxiliary risk stratification tool and should be combined with other clinical indicators for comprehensive judgment.

## Data Availability

The raw data supporting the conclusions of this article will be made available by the authors, without undue reservation.
