# Supplementary material for: Establishment and validation of a machine learning-based predictive model for in-hospital mortality risk in acute myocardial infarction patients complicated with diabetes mellitus
Source: Front Cardiovasc Med. 2026 Jun 1;13:1833723. doi: 10.3389/fcvm.2026.1833723 (PMC13265503; doi:10.3389/fcvm.2026.1833723)
Supplement: Supplementary file 1 [file Datasheet1.pdf]

Supplementary Table S1. Disease codes included in the study.

Diagnosis	Code Type	Codes
Acute myocardial infarction	ICD-9	41000,41001,41002,41010,41011,41012,41020,41021,41022,41030,41031,41032,41040,41041,41042,41050,41051,41052,41060,41061,41062,41070,41071,41072,41080,41081,41082,41090,41091,41092
	ICD-10	121,1210,12101,12102,12109,1211,12111,12119,1212,12121,12129,1213,1214,1219,121A,121A1,121A9
Old myocardial infarction	ICD-9	412
	ICD-10	1252
Diabetes Mellitus	ICD-9	25000,25001,25002,25012,25013,25040,25041,25042,25043,25050,25051,25052,25053,25060,25061,25062,25063,25070,25072,25080,25081,25082,25090,25092
	ICD-10	E1010,E1021,E1022,E10319,E1040,E1042,E1043,E1051,E10621,E1065,E109,E10649,E1110,E1121,E1122,E1136,E1139,E1140,E1142,E1143,E1151,E1152,E11610,E11621,E11622,E11628,E11649,E1165,E1169,E118,E119,E11319
malignant tumor	ICD-9	V103,V1046,V1083,V1005,1985,1977,1970,1983,V160,V1011,V1052,V1051,185,1976,V163,19889,1629
	ICD-10	Z85828,Z853,Z8546,C787,C7951,Z85038,Z800,Z85118,C786,C7931,Z803,C61,Z8551

Supplementary Table S2. Missing rate of each variable in the MIMIC-IV database.

Variables	Type variable	Validity	Missing	Proportion
Gender	category	969	0	0%
hypertension	category	969	0	0%
Heart failure	category	969	0	0%
Pulmonary disease	category	969	0	0%
Peripheral vascular disease	category	969	0	0%
Renal disease	category	969	0	0%
Liver disease	category	969	0	0%
Antiplatelet drugs	category	969	0	0%
Statins	category	969	0	0%
Anticoagulant drugs	category	969	0	0%
ACEI/ARB	category	969	0	0%
Diabetes mellitus	category	969	0	0%
Age	continue	969	0	0%

Height	continue	751	218	22.5%
Weight	continue	969	0	0%
LVEF	continue	231	738	76.16%
Temperature	continue	833	136	14%
Heart rate	continue	858	111	11.46%
Respiratory rate	continue	858	111	11.46%
SBP	continue	858	111	11.46%
DBP	continue	858	111	11.46%
WBC	continue	969	0	0%
Neutrophil count	continue	969	0	0%
Monocyte count	continue	969	0	0%
Lymphocyte count	continue	969	0	0%
RBC	continue	969	0	0%
PLT	continue	969	0	0%
CRP	continue	64	905	93.4%
Total_protein	continue	81	888	91.64%
Albumin	continue	689	280	28.9%
Creatinine	continue	969	0	0%
BUN	continue	969	0	0%
PT	continue	959	10	1.03%
APTT	continue	955	14	1.44%
INR	continue	959	10	1.03%
Fibrinogen	continue	411	558	57.59%
Thrombin	continue	2	967	99.79%
D_Dimer	continue	34	935	96.49%
cTnT	continue	758	211	21.78%
NT-proBNP	continue	136	833	85.96%
ALT	continue	855	114	11.76%
AST	continue	861	108	11.15%
ALP	continue	856	113	11.66%
Total bilirubin	continue	857	112	11.56%
Direct bilirubin	continue	62	907	93.60%
Indirect bilirubin	continue	59	910	93.91%
ck_cpk	continue	567	402	41.49%
ld_ldh	continue	663	306	31.58%
Blood glucose	continue	969	0	0%
Hemoglobin	continue	969	0	0%
Hemoglobin_a1c	continue	458	511	52.73%

Triglycerides	continue	246	723	74.61%
Total cholesterol	continue	208	761	78.53%
HDL	continue	203	766	79.05%
LDL	continue	204	765	78.95%
Potassium	continue	969	0	0%
Sodium	continue	969	0	0%
Chloride	continue	969	0	0%

Supplementary Table S3. Relevant standards or statistical parameters

Method	Standard or statistical parameters
Random Forest	max_iter=10, random_state=0
Lasso	λ=0.026
Boruta	n_iter=30, n_estimators=200

Supplementary Table S4. Hyperparameter search space of seven machine learning models.

Model	Hyperparameter search space
LR	C: [0.001, 0.01, 0.1, 1, 10, 100]; penalty: [l2]; solver: [liblinear, lbfgs]; class_weight: [None, balanced]
KNN	n_neighbors: [3, 4, 5, 6, 7, 8, 9, 10, 11, 12]; weights: [uniform, distance]; algorithm: [auto, ball_tree, kd_tree, brute]; p: [1, 2]
SVM	C: [0.01, 0.1, 0.5, 0.9, 1, 5, 10]; kernel: [linear, rbf]; gamma: [scale, auto, 0.001, 0.01, 0.1, 1]; class_weight: [None, balanced]
DT	max_depth: [2, 3, 4, 5, 6, 8, 10, None]; min_samples_split: [2, 5, 10]; min_samples_leaf: [1, 2, 4]; criterion: [gini, entropy]
LightGBM	n_estimators: [11, 50, 100, 200]; max_depth: [2, 3, 4, 5, -1]; learning_rate: [0.01, 0.05, 0.1, 0.2]; num_leaves: [7, 15, 31]; class_weight: [None, balanced]
AdaBoost	n_estimators: [11, 50, 100, 200]; learning_rate: [0.01, 0.05, 0.1, 0.5, 1.0]; algorithm: [SAMME, SAMME.R]
XGBoost	n_estimators: [11, 50, 100, 200]; max_depth: [2, 3, 4, 5]; learning_rate: [0.01, 0.05, 0.1, 0.2, 0.3]; subsample: [0.7, 0.8, 1.0]; colsample_bytree: [0.7, 0.8, 1.0]; scale_pos_weight: [1, ratio of negative to positive cases]

Supplementary Table S5. Parameters related to seven machine learning models.

Model	Optimal hyperparameters
LR	C = 1.0; penalty = l2; solver = lbfgs; class_weight = balanced; random_state = 0
KNN	n_neighbors = 6; weights = uniform; algorithm = kd_tree; p = 2
SVM	C = 0.9; kernel = rbf; gamma = scale; class_weight = balanced; probability = True; random_state = 0
DT	max_depth = 3; min_samples_split = 2; min_samples_leaf = 1; criterion = gini; random_state = 0
LightGBM	n_estimators = 11; max_depth = 3; learning_rate = 0.1; num_leaves = 31; class_weight = balanced; objective = binary
AdaBoost	n_estimators = 11; learning_rate = 1.0; algorithm = SAMME.R; random_state = 0
XGBoost	n_estimators = 11; max_depth = 3; learning_rate = 0.3; subsample = 1.0; colsample_bytree = 1.0; scale_pos_weight = 1; objective = binary:logistic; use_label_encoder = False; eval_metric = error

Supplementary Table S6. Baseline characteristics between the survival group and the non-survival group in the internal validation set.

Variables	Total(N=291)	Survival(N=251)	Non-survival(N=40)	*P* value
**Demographics**				
Age(years)	70.3 (62.1-78.8)	70 (61.7-78)	73.3 (65.1-82.5)	0.074
BMI(Kg/m^2)	29 (25.5-34.1)	28.9 (25.8-34.2)	29.4 (24.8-33.9)	0.948
Gender(n,%)				0.833
female	94 (32.3%)	80 (31.9%)	14 (35%)	
male	197 (67.7%)	171 (68.1%)	26 (65%)	
**Vital signs**				
Temperature(℃)	36.6 (36.4-36.9)	36.6 (36.4-36.9)	36.8 (36.4-37.1)	0.089
Heart rate(bpm)	85 (78-96)	85 (78-94.5)	88.5 (81.5-106.2)	0.048
Respiratory rate(bpm)	18 (16-22)	18 (16-22)	21 (18.8-27.2)	<0.001
SBP(mmHg)	123 (108-137)	124 (109-138)	116.5 (99-130.5)	0.055
DBP(mmHg)	64.7 (54.5-74)	65 (55-73)	59.5 (48.8-78)	0.479
**Laboratory measurements**				
WBC(10^9/L)	11.2 (8.1-14.8)	10.9 (7.9-14)	14 (10.8-19.5)	<0.001
NEUT(10^9/L)	9.7 (6.6-13.1)	9.3 (6.4-12.5)	12.7 (9.1-15.6)	0.002
LYMPH(10^9/L)	1.3 (0.8-2)	1.3 (0.8-2)	0.9 (0.7-2)	0.156
MONO(10^9/L)	0.7 (0.4-1)	0.6 (0.4-0.9)	0.9 (0.6-1.1)	0.005
RBC(10^{12}/L)	3.7 (3.1-4.3)	3.7 (3.1-4.4)	3.4 (2.9-4.1)	0.075
PLT(10^9/L)	205 (156-261)	204 (157-256.5)	214.5 (148-298)	0.604
SII	1436.4 (645.5-2783.9)	1359.9 (633-2652.9)	2239.2 (971.6-5381)	0.028
PLR	166.9 (93.2-276.6)	163.1 (92.4-269.3)	193.9 (101.6-345.1)	0.282
NLR	7.2 (3.6-13.3)	7 (3.6-13.1)	9.9 (4.9-19.6)	0.025
Hb(g/L)	112 (93-131)	112 (95-133)	106 (88.2-117.8)	0.018
PT(s)	13.6 (12.4-15.8)	13.4 (12.2-15.4)	14.9 (13.8-16.7)	<0.001
APTT(s)	35.8 (29.1-54.1)	35.8 (29.4-54.5)	36.2 (28.9-48.5)	0.951
INR	1.2 (1.1-1.4)	1.2 (1.1-1.4)	1.4 (1.2-1.5)	<0.001
AST(IU/L)	50 (24-115.5)	43 (23-97.5)	108 (44.5-260.8)	<0.001
ALT(IU/L)	28 (18-63)	27.4 (18-58)	44.5 (21.5-144.2)	0.031
Albumin(g/L)	34 (30.7-37)	34.6 (31.2-37)	29 (26.8-32)	<0.001
ALP(IU/L)	86 (62-111.5)	85.3 (62-111.1)	88.5 (62-120.8)	0.609
TBiL(mg/dL)	0.5 (0.4-0.8)	0.5 (0.4-0.8)	0.6 (0.3-1.6)	0.057
Creatinine(mg/dL)	1.4 (0.9-2.4)	1.3 (0.9-2.2)	1.8 (1.3-3)	0.007
BUN(mg/dL)	28 (16-48)	26 (16-47)	39.5 (24.8-49.8)	0.009
cTnT(ng/mL)	0.5 (0.2-1.3)	0.5 (0.2-1.1)	1.1 (0.2-2.4)	0.061
Blood glucose(mg/dL)	181 (129.5-276.5)	182 (131-262)	174.5 (119.5-333.5)	0.536

Potassium(mmol/L)	4.2 (3.9-4.7)	4.2 (4-4.7)	4.2 (3.7-4.8)	0.578
Sodium(mmol/L)	138 (135-140.5)	138 (135-140.5)	138 (133-140.2)	0.629
Chloride(mmol/L)	102 (98-105)	102 (99-105)	102.5 (97-106)	0.789
**Comorbidities**				
Hypertension(n,%)				0.878
No	197 (67.7%)	169 (67.3%)	28 (70%)	
Yes	94 (32.3%)	82 (32.7%)	12 (30%)	
Heart failure(n,%)				0.401
No	117 (40.2%)	98 (39%)	19 (47.5%)	
Yes	174 (59.8%)	153 (61%)	21 (52.5%)	
Pulmonary disease(n,%)				0.079
No	218 (74.9%)	193 (76.9%)	25 (62.5%)	
Yes	73 (25.1%)	58 (23.1%)	15 (37.5%)	
PVD(n,%)				0.499
No	248 (85.2%)	212 (84.5%)	36 (90%)	
Yes	43 (14.8%)	39 (15.5%)	4 (10%)	
Renal disease(n,%)				0.151
No	165 (56.7%)	147 (58.6%)	18 (45%)	
Yes	126 (43.3%)	104 (41.4%)	22 (55%)	
Liver disease(n,%)				0.536
No	268 (92.1%)	232 (92.4%)	36 (90%)	
Yes	23 (7.9%)	19 (7.6%)	4 (10%)	
**Treatments**				
Antiplatelet drugs(n,%)				0.027
No	60 (20.6%)	46 (18.3%)	14 (35%)	
Yes	231 (79.4%)	205 (81.7%)	26 (65%)	
Statins(n,%)				0.915
No	242 (83.2%)	208 (82.9%)	34 (85%)	
Yes	49 (16.8%)	43 (17.1%)	6 (15%)	
Anticoagulant drugs(n,%)				0.947
No	41 (14.1%)	36 (14.3%)	5 (12.5%)	
Yes	250 (85.9%)	215 (85.7%)	35 (87.5%)	
ACEI/ARB(n,%)				0.398
No	261 (89.7%)	223 (88.8%)	38 (95%)	
Yes	30 (10.3%)	28 (11.2%)	2 (5%)	
DM(n,%)				0.764
No	121 (41.6%)	103 (41%)	18 (45%)	
Yes	170 (58.4%)	148 (59%)	22 (55%)	

Note. BMI:body mass index , SBP:systolic blood pressure, DBP:diastolic blood pressure, WBC:white blood cell count, NEUT:neutrophil count, LYMPH:lymphocyte count, MONO:monocyte count, RBC:red blood cell count, PLT:platelet count, Hb:hemoglobin, SII:systemic immune-inflammation index, PLR:platelet-to-lymphocyte ratio, NLR:neutrophil-to-lymphocyte ratio, PT:prothrombin time, APTT:activated partial thromboplastin time, INR:international normalized ratio, AST:aspartate aminotransferase, ALT:alanine aminotransferase ALP:alkaline phosphatase, TBiL:total bilirubin, BUN:blood urea nitrogen, cTnT:cardiac troponin T, PVD:peripheral vascular disease, ACEI/ARB:Angiotensin-Converting Enzyme Inhibitor/Angiotensin II Receptor Blocker; DM: Diabetes Mellitus.

Supplementary Table S7. Baseline characteristics between the survival group and the non-survival group in the external validation set.

Variables	Total(N=477)	Survival(N=386)	Non-survival(N=91)	*P* value
**Demographics**				
Age(years)	64 (55-73)	61.5 (55-72)	71 (62.5-76.5)	<0.001
BMI(Kg/m^2)	24.6 ± 3.2	24.7 ± 3.3	24.2 ± 2.9	0.151
Gender(n,%)				0.588
female	123 (25.8%)	97 (25.1%)	26 (28.6%)	
male	354 (74.2%)	289 (74.9%)	65 (71.4%)	
**Vital signs**				
Temperature(°C)	36.5 (36.5-36.6)	36.5 (36.5-36.6)	36.5 (36.5-36.6)	0.598
Heart rate(bpm)	80 (72-89)	79.5 (72-88)	86 (77.5-95.5)	<0.001
Respiratory rate(bpm)	20 (20-21)	20 (20-21)	21 (20-22)	0.007
SBP(mmHg)	127.8 ± 21.5	128.8 ± 21.3	123.5 ± 21.9	0.038
DBP(mmHg)	78.8 ± 14.6	79 ± 14.5	78 ± 15.3	0.561
**Laboratory measurements**				
WBC(10^9/L)	8.9 (7-11.5)	8.3 (6.7-10.4)	12.2 (9.4-13.7)	<0.001
NEUT(10^9/L)	6.7 (4.7-9.2)	6 (4.4-8.3)	9.8 (7.7-11.7)	<0.001
LYMPH(10^9/L)	1.4 (1-1.9)	1.5 (1.1-1.9)	1.3 (0.9-1.7)	0.049
MONO(10^9/L)	0.5 (0.4-0.7)	0.5 (0.4-0.6)	0.6 (0.4-0.8)	0.009
RBC(10^{12}/L)	4.4 (4-4.9)	4.5 (4-4.9)	4.2 (3.6-4.6)	<0.001
PLT(10^9/L)	196 (157-241)	197 (160-241.8)	186 (149.5-236.5)	0.185
SII	833.6(518.3-1534.4)	761(490.8-1377.7)	1411.2 (764-2235.6)	<0.001
PLR	134.7 (96.2-190.4)	132.9 (96.2-189.2)	141.4 (96.3-195.5)	0.400
NLR	4.5 (2.8-7.8)	3.9 (2.6-7)	7.3 (4.8-11.7)	<0.001
Hb(g/L)	133 (119-146)	135 (121-147.8)	123 (110-136.5)	<0.001
PT(s)	12.7 (12-13.6)	12.6 (11.9-13.5)	13 (12.2-15.2)	0.002
APTT(s)	33.8 (29.6-40.3)	33.6 (29.8-40)	34 (29.1-40.8)	0.817
INR	1 (0.9-1.1)	1 (0.9-1.1)	1.1 (1-1.2)	<0.001
AST(IU/L)	40 (22-115)	35.5 (22-91.5)	68 (25-196)	<0.001
ALT(IU/L)	30 (18-53)	30 (18-50.8)	33 (18-60.5)	0.143
Albumin(g/L)	40.2 (37.3-42.7)	40.4 (37.7-42.8)	38.7 (35.8-42)	0.001
ALP(IU/L)	81 (67-101)	82 (67-100)	80 (66-105.5)	0.935
TBiL(mg/dL)	0.7 (0.5-0.9)	0.7 (0.5-0.9)	0.7 (0.5-1.1)	0.269
Creatinine(mg/dL)	0.9 (0.7-1.2)	0.9 (0.7-1)	1.2 (0.9-1.7)	<0.001
BUN(mg/dL)	17.6 (13.7-23.6)	17.1 (13.3-22.1)	23 (15.9-36.2)	<0.001
cTnT(ng/mL)	0.5 (0.1-1.6)	0.3 (0.1-1.4)	0.7 (0.2-2.9)	0.001
Blood glucose(mg/dL)	176.9 (142-234)	176.9 (142.9-224.1)	179.8(139.5-261.2)	0.239

Potassium(mmol/L)	3.8 (3.5-4.1)	3.8 (3.5-4.1)	4 (3.6-4.4)	0.001
Sodium(mmol/L)	137.5 (134-139.8)	137.9 (135-140.1)	136 (132-138.4)	<0.001
Chloride(mmol/L)	104 (101-106.5)	104 (101.5-106.9)	103 (99.8-105.2)	0.004
**Comorbidities**				
Hypertension(n,%)				0.785
No	171 (35.8%)	140 (36.3%)	31 (34.1%)	
Yes	306 (64.2%)	246 (63.7%)	60 (65.9%)	
Heart failure(n,%)				<0.001
No	265 (55.6%)	250 (64.8%)	15 (16.5%)	
Yes	212 (44.4%)	136 (35.2%)	76 (83.5%)	
Pulmonary disease(n,%)				<0.001
No	290 (60.8%)	262 (67.9%)	28 (30.8%)	
Yes	187 (39.2%)	124 (32.1%)	63 (69.2%)	
PVD(n,%)				0.001
No	381 (79.9%)	320 (82.9%)	61 (67%)	
Yes	96 (20.1%)	66 (17.1%)	30 (33%)	
Renal disease(n,%)				<0.001
No	403 (84.5%)	344 (89.1%)	59 (64.8%)	
Yes	74 (15.5%)	42 (10.9%)	32 (35.2%)	
Liver disease(n,%)				0.103
No	383 (80.3%)	316 (81.9%)	67 (73.6%)	
Yes	94 (19.7%)	70 (18.1%)	24 (26.4%)	
**Treatments**				
Antiplatelet drugs(n,%)				0.181
No	27 (5.7%)	25 (6.5%)	2 (2.2%)	
Yes	450 (94.3%)	361 (93.5%)	89 (97.8%)	
Statins(n,%)				0.182
No	8 (1.7%)	5 (1.3%)	3 (3.3%)	
Yes	469 (98.3%)	381 (98.7%)	88 (96.7%)	
Anticoagulant drugs(n,%)				0.419
No	98 (20.5%)	76 (19.7%)	22 (24.2%)	
Yes	379 (79.5%)	310 (80.3%)	69 (75.8%)	
ACEI/ARB(n,%)				0.544
No	304 (63.7%)	243 (63%)	61 (67%)	
Yes	173 (36.3%)	143 (37%)	30 (33%)	
DM(n,%)				<0.001

No	263 (55.1%)	247 (64%)	16 (17.6%)
Yes	214 (44.9%)	139 (36%)	75 (82.4%)

Note. BMI:body mass index , SBP:systolic blood pressure, DBP:diastolic blood pressure, WBC:white blood cell count, NEUT:neutrophil count, LYMPH:lymphocyte count, MONO:monocyte count, RBC:red blood cell count, PLT:platelet count, Hb:hemoglobin, SII:systemic immune-inflammation index, PLR:platelet-to-lymphocyte ratio, NLR:neutrophil-to-lymphocyte ratio, PT:prothrombin time, APTT:activated partial thromboplastin time, INR:international normalized ratio, AST:aspartate aminotransferase, ALT:alanine aminotransferase ALP:alkaline phosphatase, TBiL:total bilirubin, BUN:blood urea nitrogen, cTnT:cardiac troponin T, PVD:peripheral vascular disease, ACEI/ARB:Angiotensin-Converting Enzyme Inhibitor/Angiotensin II Receptor Blocker; DM: Diabetes Mellitus.

Supplementary Table S8. Common variables screened by the two methods

Methods	Variables	Common variables
Lasso	Heart rate、Respiratory rate、NEUT、MONO、PLT、NLR、INR、Albumin、TBiL、BUN	Heart rate、NEUT、MONO、NLR、Albumin、TBiL、BUN
Boruta	Heart rate、NEUT、LYMPH、MONO、SII、NLR、PT、Albumin、TBiL、BUN	

Note. NEUT:neutrophil count, LYMPH:lymphocyte count, MONO:monocyte count, PLT:platelet count, SII:systemic immune-inflammation index, NLR:neutrophil-to-lymphocyte ratio, PT:prothrombin time, INR:international normalized ratio, TBiL:total bilirubin, BUN:blood urea nitrogen.

Supplementary Table S9. Results of each model in the training set.

**Model**	**AUC(95%CI)**	**Accuracy**	**Sensitivity**	**Specificity**	**PPV**	**NPV**	**F1 score**	**Cutoff**
LR	0.779(0.725-0.832)	0.836	0.620	0.883	0.536	0.914	0.575	0.178
KNN	0.859(0.831-0.887)	0.804	0.628	0.842	0.463	0.912	0.533	0.167
DT	0.801(0.759-0.843)	0.836	0.413	0.928	0.556	0.897	0.474	0.258
XGBoost	0.907(0.876-0.939)	0.888	0.752	0.917	0.664	0.945	0.705	0.281
LightGBM	0.875(0.842-0.908)	0.804	0.818	0.801	0.471	0.953	0.598	0.534

AdaBoost	0.838(0.802-0.874)	0.819	0.653	0.855	0.494	0.919	0.562	0.473
SVM	0.873(0.836-0.887)	0.779	0.843	0.765	0.438	0.957	0.576	0.195

Supplementary Table S 10. Calibration metrics of the seven models in internal and external validation sets.

Model	Calibration intercept	calibration slope	Brier score	observed-to-expected ratio
**Internal**				
LR	-0.42	0.58	0.105	0.78
KNN	-0.06	0.93	0.088	0.97
SVM	-0.28	0.72	0.096	0.86
DT	0.03	0.86	0.091	1.02
LightGBM	-0.68	0.46	0.116	0.67
AdaBoost	-0.61	0.50	0.113	0.71
XGBoost	-0.03	0.96	0.087	0.99
**External**				
LR	-0.78	0.42	0.151	0.63
KNN	-0.26	0.76	0.128	0.85
SVM	0.34	0.61	0.139	1.20
DT	0.04	0.98	0.118	1.03
LightGBM	-0.62	0.54	0.142	0.72
AdaBoost	-0.58	0.56	0.140	0.75
XGBoost	0.02	1.01	0.116	1.01

Supplementary Table S11. Clinical cutoff values of continuous variables.

Continuous Predictor	Optimal Cutoff Value	AUC(95%CI)	Sensitivity	Specificity	Youden's Index
Training Set					
HR	≥93bpm	0.598(0.540-0.655)	0.521	0.709	0.230
NEUT	≥11.48×10^9/L	0.648(0.591-0.705)	0.545	0.704	0.249
MONO	≥0.77×10^9/L	0.634(0.577-0.692)	0.570	0.673	0.243
NLR	≥8.527	0.708(0.653-0.763)	0.719	0.657	0.376
Albumin	≤30.84g/L	0.709(0.653-0.764)	0.521	0.801	0.321
TBiL	≥0.711mg/mL	0.666(0.609-0.722)	0.521	0.741	0.262
BUN	≥32mg/dL	0.661(0.604-0.717)	0.603	0.623	0.226
Internal Validation Set					
HR	≥93bpm	0.591(0.492-0.691)	0.410	0.698	0.109

NEUT	≥11.48×10^9/L	0.728(0.634-0.822)	0.667	0.722	0.389
MONO	≥0.77×10^9/L	0.723(0.628-0.818)	0.718	0.683	0.400
NLR	≥8.527	0.717(0.621-0.812)	0.744	0.611	0.355
Albumin	≤30.84g/L	0.844(0.766-0.923)	0.692	0.810	0.502
TBiL	≥0.711mg/mL	0.653(0.554-0.752)	0.487	0.746	0.233
BUN	≥32mg/dL	0.680(0.582-0.777)	0.667	0.615	0.282
External Validation Set					
HR	≥93bpm	0.663(0.597-0.728)	0.341	0.829	0.170
NEUT	≥11.48×10^9/L	0.807(0.750-0.863)	0.319	0.927	0.246
MONO	≥0.77×10^9/L	0.638(0.572-0.705)	0.308	0.878	0.186
NLR	≥8.527	0.768(0.708-0.828)	0.484	0.839	0.323
Albumin	≤30.84g/L	0.668(0.603-0.734)	0.033	0.977	0.010
TBiL	≥0.711mg/mL	0.680(0.582-0.777)	0.667	0.615	0.282
BUN	≥32mg/dL	0.675(0.609-0.740)	0.297	0.925	0.222

Supplementary Table S12. Results of subgroup analysis.

**Cohort**	**Model**	**AUC(95%CI)**	**Accuracy**	**Sensitivity**	**Specificity**	**PPV**	**NPV**	**F1 score**
Internal	Age<65	0.869(0.812-0.926)	0.794	0.846	0.786	0.779	0.971	0.524
	Age≥65	0.849(0.771-0.928)	0.876	0.718	0.901	0.728	0.954	0.609
External	Age<65	0.840(0.800-0.880)	0.711	0.879	0.671	0.386	0.959	0.386
	Age≥65	0.822(0.774-0.870)	0.765	0.769	0.764	0.635	0.934	0.556
Internal	Male	0.860(0.827-0.893)	0.786	0.793	0.785	0.444	0.946	0.444
	Female	0.847(0.814-0.879)	0.745	0.810	0.731	0.395	0.947	0.395
External	Male	0.816(0.779-0.852)	0.739	0.769	0.732	0.384	0.936	0.384
	Female	0.824(0.785-0.863)	0.751	0.769	0.747	0.397	0.937	0.397

Supplementary Table S13. Comparison of results based on XGBoost with and without SMOTE

Index	Internal		External	
	No-Smote	Smote	No-Smote	Smote
AUC(95%CI)	0.876(0.818-0.933)	0.873(0.815-0.931)	0.846(0.808-0.885)	0.845(0.807-0.884)
Accuracy	0.749	0.684	0.740	0.736
Sensitivity	0.897	0.923	0.791	0.769
Specificity	0.726	0.647	0.728	0.728
PPV	0.337	0.288	0.407	0.400
NPV	0.979	0.982	0.937	0.930

F1 score	0.490	0.439	0.537	0.526

Supplementary Table S14. Results of each model in the internal validation set and in the external validation set without the inclusion of SMOTE.

**Model**	**AUC**	**Accuracy**	**Sensitivity**	**Specificity**	**PPV**	**NPV**	**F1 score**
**Internal**							
LR	0.633	0.649	0.692	0.643	0.231	0.931	0.190
KNN	0.746	0.808	0.538	0.849	0.356	0.922	0.429
DT	0.777	0.749	0.718	0.754	0.311	0.945	0.434
XGBoost	0.876	0.749	0.897	0.726	0.337	0.979	0.490
LightGBM	0.862	0.742	0.872	0.722	0.327	0.973	0.476
AdaBoost	0.838	0.814	0.718	0.829	0.394	0.950	0.509
SVM	0.835	0.739	0.846	0.722	0.320	0.968	0.465
External							
LR	0.771	0.748	0.736	0.751	0.411	0.924	0.528
KNN	0.741	0.797	0.505	0.865	0.469	0.881	0.487
DT	0.765	0.602	0.923	0.526	0.315	0.967	0.469
XGBoost	0.846	0.740	0.791	0.728	0.407	0.937	0.537
LightGBM	0.833	0.742	0.747	0.741	0.405	0.926	0.525
AdaBoost	0.802	0.686	0.791	0.661	0.355	0.931	0.490
SVM	0.833	0.734	0.813	0.715	0.402	0.942	0.538

Note. LR:Logistic Regression, KNN:K-Nearest Neighbors, SVM:Support Vector Machine, DT:Decision Tree, LightGBM:Light Gradient Boosting Machine, AdaBoost:Adaptive Boosting, XGBoost:eXtreme Gradient Boosting, PPV:Positive Predictive Value, NPV:Negative Predictive Value.

Supplementary Table S15. Hosmer－Lemeshow test results for 7 models without the inclusion of SMOTE.

	**LR**	**KNN**	**SVM**	**DT**	**LightGBM**	**AdaBoost**	**XGBoost**	
Train	<0.001	0.963	<0.001	<0.001	<0.001	<0.001	0.123	*P* value
Internal	0.004	0.005	0.022	<0.001	<0.001	<0.001	0.177	*P* value
External	<0.001	0.016	<0.001	<0.001	<0.001	<0.001	0.129	*P* value

Note. LR:Logistic Regression, KNN:K-Nearest Neighbors, SVM:Support Vector Machine, DT:Decision Tree, LightGBM:Light Gradient Boosting Machine, AdaBoost:Adaptive Boosting, XGBoost:eXtreme Gradient Boosting.

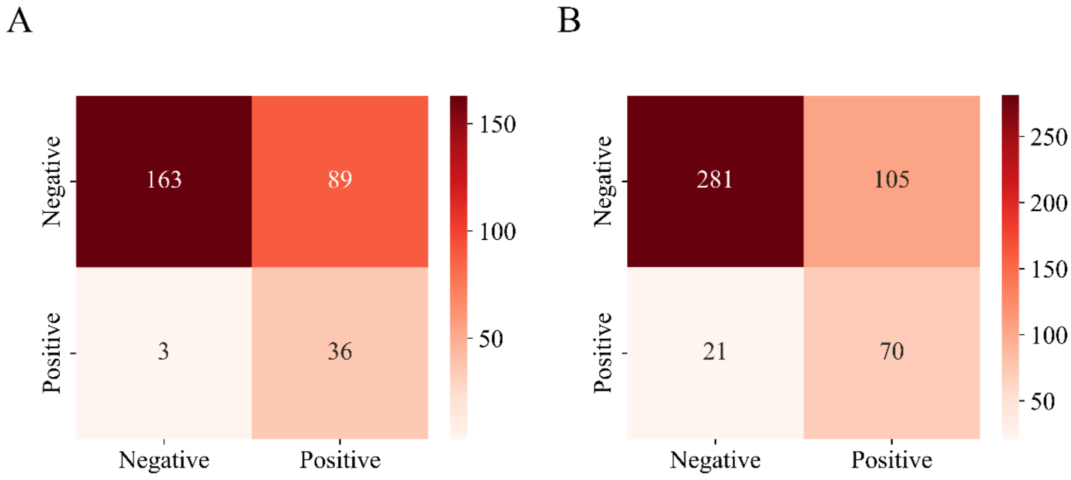

Supplementary Figure S1. Confusion matrices of the XGBoost model. (A) Internal validation set. (B) External validation set.

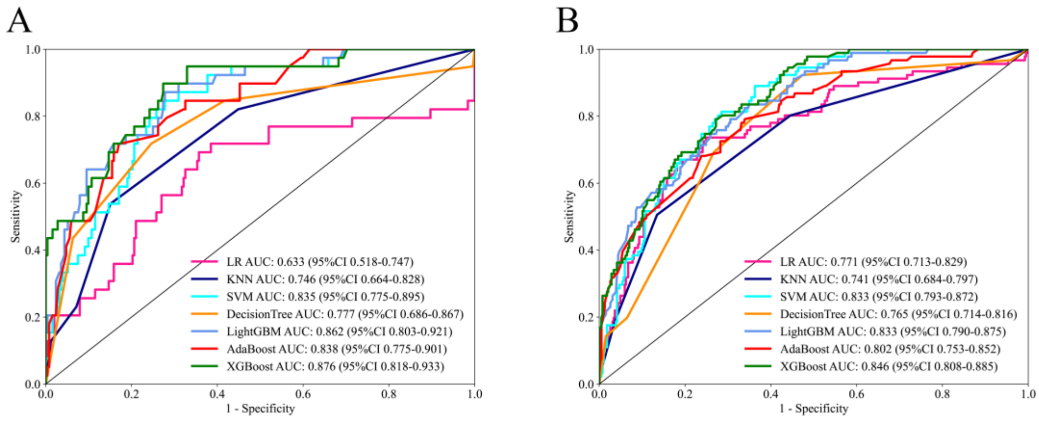

Supplementary Figure S2. Comparison of ROC for each machine learning modelwithout the inclusion of SMOTE. (A) Internal validation set. (B) External validation set.

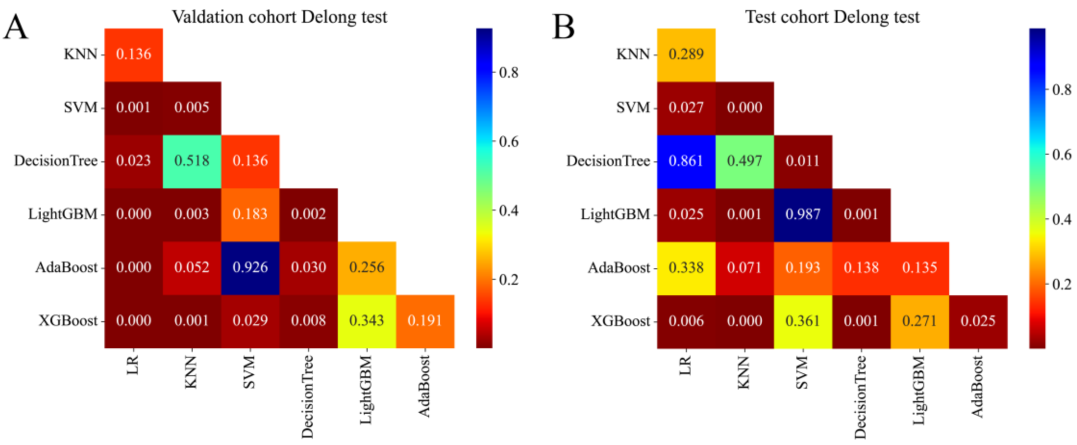

Supplementary Figure S 3. Comparison of DeLong test for each machine learning model without the inclusion of SMOTE. (A) Internal validation set. (B) External validation set.

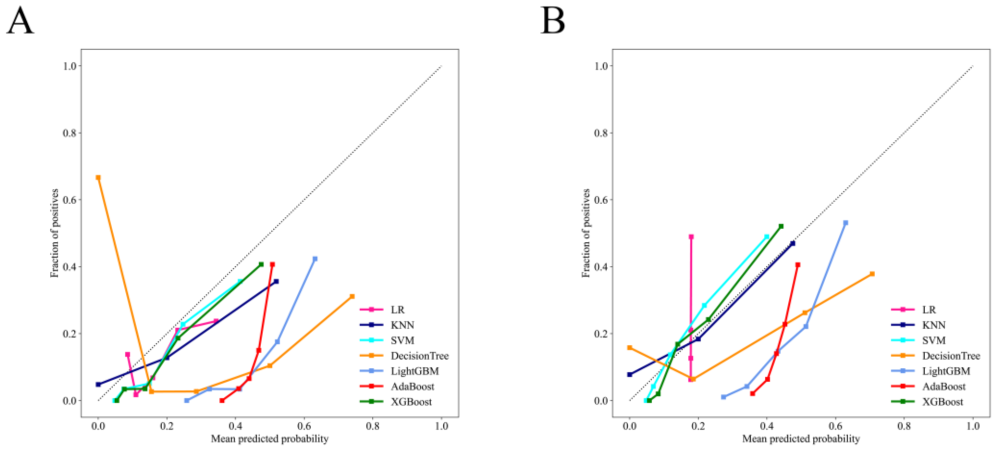

Supplementary Figure S4. Comparison of calibration curves for each machine learning without the inclusion of SMOTE. (A)Internal validation set. (B) External validation set.

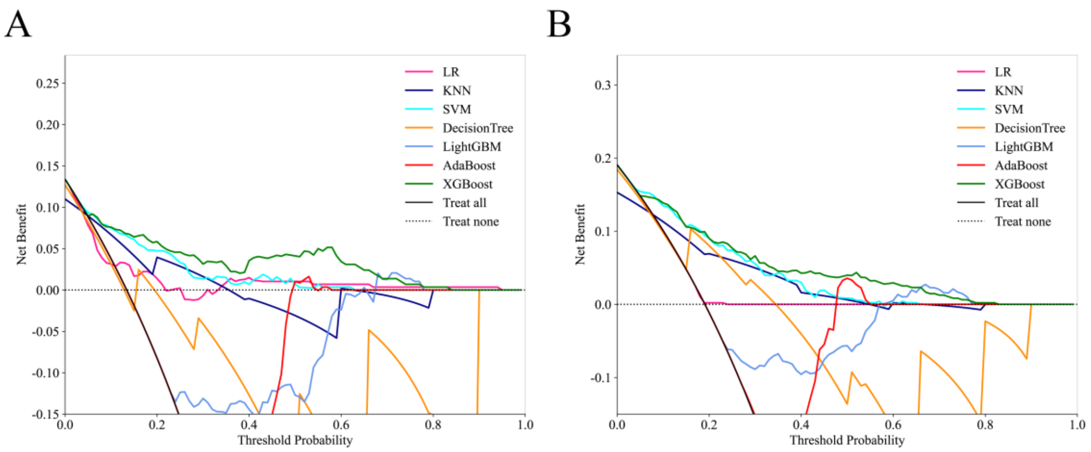

Supplementary Figure S5. Comparison of DCA for each machine learning without the inclusion of SMOTE. (A) Internal validation set. (B) External validation set.
